# Reinstating Niche Failure in Diabetic Cranial Defects via Chronotaxic Signal‐Amplifying Fluidic Biomimetic Hydrogel

**DOI:** 10.1002/advs.202516398

**Published:** 2025-10-15

**Authors:** Yingji Mao, Yu Chen, Runlin Fan, Pengzhen Zhuang, Hongbo Zhang, Pinghui Zhou

**Affiliations:** ^1^ Department of Orthopedics Anhui Province Key Laboratory of Tissue Transplantation The First Affiliated Hospital of Bengbu Medical University Bengbu 233004 China; ^2^ Anhui Provincial Key Laboratory of Tumor Evolution and Intelligent Diagnosis and Treatment Anhui Engineering Research Center for Neural Regeneration Technology and Medical New Materials School of Life Sciences Bengbu Medical University Bengbu 233030 China; ^3^ Pharmaceutical Sciences Laboratory Faculty of Science and Engineering Åbo Akademi University Turku 20520 Finland; ^4^ Turku Bioscience Centre University of Turku and Åbo Akademi University Turku 20520 Finland

**Keywords:** biomimetic hydrogel, cell recruitment, diabetic cranial defects, osteogenesis, signal amplification

## Abstract

Cranial stem cell niches (SCNs) are intrinsically scarce and hypoactive, and, exacerbated by chronic inflammation in diabetes, lead to niche failure and regenerative deficit after injury. Herein, an in situ moldable fluidic biomimetic niche (GelSSO/PDA@SDF) is developed as a chronotaxic signal amplifier to enhance SCN abundance and activity, aiming to restore autonomous regeneration. This biomimetic niche integrates PDA@SDF nanoparticles and a GelSSO hydrogel precursor, synthesized via dopamine self‐polymerization/protein coupling and sequential methacrylation/sequence‐specific oligodeoxynucleotide (SSO) grafting, respectively. Photocrosslinked GelSSO/PDA@SDF can preferentially and sustainably release PDA@SDF nanoparticles to trigger early‐phase signal amplification, characterized by SDF‐1α/CXCR4‐mediated recruitment of endothelial and mesenchymal progenitors, vascular niche activation driving AKT‐dependent angiogenesis, and suppressed M1 macrophage dominance. Progressive hydrogel degradation initiates the secondary signal amplification phase, in which prolonged SSO release creates a transcriptionally active osteogenic niche for MAPK/ERK‐induced osteogenesis. In vivo, the in situ structured GelSSO/PDA@SDF conformed to defect geometry, promoting the early establishment of an immunologically favorable, progenitor‐enriched niche through local immunomodulation and endogenous cell homing, followed by successive activation of vascular and osteogenic niches, ultimately achieving diabetic cranial vascularized bone regeneration. Thus, this chronotaxic signal‐amplifying biomimetic niche offers a versatile strategy for restoring autonomous regeneration in the diabetic cranium and other poorly regenerative tissues.

## Graphical abstract

An in situ moldable GelSSO/PDA@SDF hydrogel functions as a chronotaxic signal‐amplifying biomimetic niche to restore regenerative failure in diabetic cranial defects. By sequentially releasing SDF‐1α‐loaded PDA nanoparticles and osteoinductive oligodeoxynucleotides, it orchestrates immunomodulation, progenitor recruitment, angiogenesis, and osteogenesis, ultimately achieving vascularized cranial bone regeneration and offering a versatile strategy for poorly regenerative tissues.

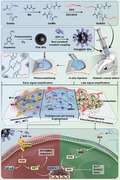

## 1 Introduction

Stem cell niches (SCNs) are specialized microenvironments that integrate multidimensional signals to preserve stemness, direct differentiation, and coordinate repair, thereby determining the regenerability of tissues.^[^ ^1^, ^2^, ^3^ ^]^ This capacity varies hierarchically across tissues and anatomy owing to the spatial heterogeneity of SCNs. From a tissue perspective, the skin and liver harbor numerous, broadly distributed, and highly injury‐responsive SCNs that enable rapid restoration of structure and function. In contrast, the myocardium and central nervous system, characterized by SCN scarcity and hypoactivity, are poorly regenerative, leading to functionally limited scarring.^[^ ^4^, ^5^ ^]^ Anatomically, marrow‐rich endosteal and periosteal SCNs in the femur support structural and mechanical healing, while the cranium, lacking such supportive SCNs, struggles to bridge defects.^[^ ^6^, ^7^ ^]^ Beyond these baseline constraints, pathological conditions such as diabetes further deplete SCN abundance and activity via chronic inflammation, exacerbating the regenerative deficit, especially in tissues where SCNs are already scarce.^[^ ^8^, ^9^ ^]^ Thus, enhancing SCN abundance and activity in intrinsically and pathologically disadvantaged tissues is crucial for restoring their regenerability and facilitating effective repair.

To address intrinsic SCN limitations, two principal biomimetic niche strategies have emerged: integrating exogenous stem cells with functional biomaterials and designing cell‐free systems. In exogenous strategies, biomimetic niches deliver targeted signals to enhance the differentiation of transplanted cells and sustain tissue repair.^[^ ^10^, ^11^ ^]^ For instance, conductive cardiac patches composed of carbon nanotubes or polypyrrole enhance electrophysiological signals in cardiac SCNs, promoting the coupling and maturation of grafted cardiomyocytes and thereby improving myocardial regenerability.^[^ ^12^, ^13^, ^14^ ^]^ In our previous work, neural SCNs engineered by co‐encapsulating bone marrow‐ and neural‐derived stem cells in a 3D hydrogel provided synergistic mechanical and paracrine signals that enhanced neurogenic potential, suppressing fibrosis and promoting spinal cord repair.^[^ ^15^ ^]^ Cell‐free approaches instead endow biomimetic niches with additional chemotactic signals to mobilize endogenous stem cells from distal reservoirs, thereby compensating for insufficient cellular resources.^[^ ^16^, ^17^, ^18^ ^]^ Hassan et al. developed an injectable hydrogel niche that sustainably released stromal cell‐derived factor‐1α (SDF‐1α) and oxygen in myocardial infarction, fostering progenitor cell homing and survival to improve cardiac function.^[^ ^19^ ^]^ Our previous study found that nanofibrous membrane‐based osteogenic SCNs enhanced angiogenesis during cranial bone repair, since the SDF‐1α released from the niche recruited endothelial progenitor cells (EPCs) and activated vascular niche signals.^[^ ^20^ ^]^ Although cell‐free strategies surpass exogenous ones in cell availability and cell‐type diversity, their indiscriminate chemotaxis also drives macrophage influx.^[^ ^21^, ^22^, ^23^, ^24^ ^]^ The macrophage transition from pro‐inflammatory (M1) to reparative (M2) usually occurs as injury signals subside, but is blocked by aberrant signals in diabetes, perpetuating inflammation that undermines the SCN‐supporting efficacy of biomimetic niches.^[^ ^8^, ^25^, ^26^, ^27^ ^]^ As niches supply synchronized chemotactic and angiogenic signals, incorporating time‐locked immunomodulatory signals is therefore crucial to sustain M1‐to‐M2 transitions for niche‐mediated regeneration.

Diabetic cranial defects are highly prevalent and carry high postoperative failure risks, imposing substantial healthcare pressure and compromising patient prognosis and quality of life.^[^ ^28^, ^29^ ^]^ Confronted with the niche failure caused by diabetes‐related pathology and cranial unique anatomy, achieving functional bone regeneration remains a formidable challenge. Although niche‐mediated inflammatory control and vascular establishment offer early support for retaining homed stem cells, it is the sustained advancement of their osteogenic differentiation by the niche within the inducible signal‐deficient cranium that ultimately dictates repair outcomes.^[^ ^30^ ^]^ Existing osteoconductive materials, such as hydroxyapatite and bioactive glass, act as passive niches, relying on their intrinsic properties, but suffer from limited inducibility, poor tunability, and a lack of biospecificity.^[^ ^31^, ^32^, ^33^ ^]^ The functional coupling of responsive materials with external field stimuli, such as electromagnetic fields or mechanical stress, can simulate physiological loading fields to activate the osteogenicity of niches. However, energy attenuation due to cranial shielding weakens the precise modulation of deeper niches.^[^ ^34^, ^35^, ^36^ ^]^ Thus, enhancing the inductive efficacy of biomimetic niches through endogenous biosignals could provide a more definitive and sustained differentiation directive for stem cells. DNA, composed of deoxyribonucleotides, is the primary carrier of genetic information. Its sequence diversity confers both breadth and precision in regulating cellular responses.^[^ ^37^, ^38^ ^]^ Therefore, deoxyribonucleotide chains encoding specific sequences can be embedded into biomimetic niches as genetic signals, directly modulating intracellular pathways to precisely control stem cell differentiation at the transcriptional level. Additionally, hydrogels are ideal matrices for constructing biomimetic niches, as their injectable plasticity enables homogeneous signal distribution while in situ adapting to geometric defects.^[^ ^11^, ^39^, ^40^ ^]^

**Scheme 1:**
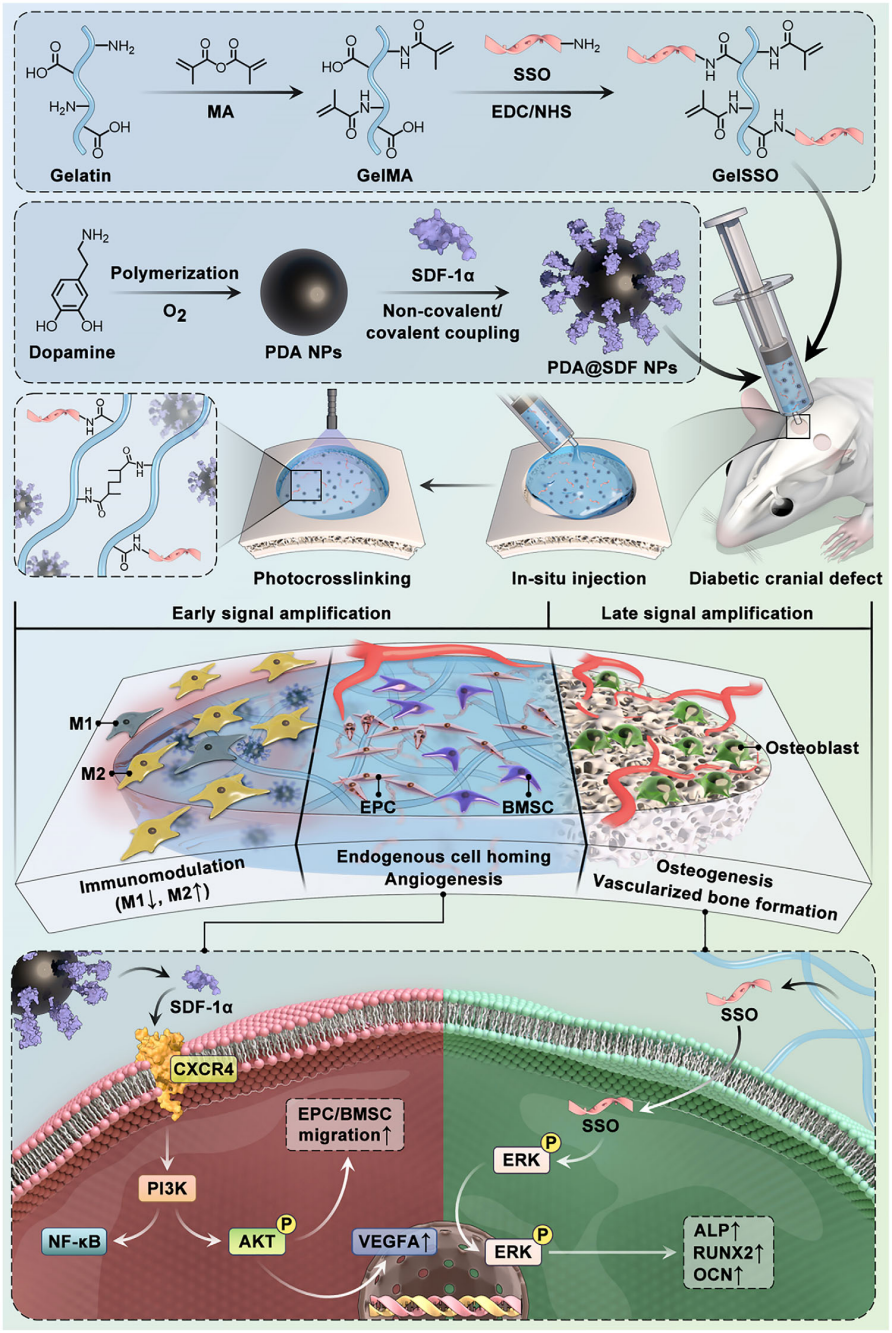
Schematic illustration of the fabrication of the GelSSO/PDA@SDF fluidic biomimetic niche and its chronotaxic signal amplification mechanism for enhanced autonomous regeneration in diabetic cranial defects.

The exertion of tissue regenerability is constrained by the complicated heterogeneity of SCNs at anatomical scales and physio‐pathological states. In this study, based on the pathological characteristics of diabetic cranial SCNs, an in situ moldable fluidic biomimetic niche was innovatively designed as a “chronotaxic signal amplifier,” augmenting the abundance and activity of local niches while mitigating cumulative inflammatory insults, thereby eliciting the autonomous regenerative potential of injured cranial bone (**Scheme** **1**). Specifically, methacrylic anhydride (MA) and sequence‐specific oligodeoxynucleotide (SSO) chains were covalently grafted onto the gelatin backbone via sequential chemical modification, obtaining a GelSSO hydrogel precursor with photocurability and genetic programmability. In addition, polydopamine nanoparticles (PDA NPs) synthesized by oxidative self‐polymerization were employed as efficient carriers for SDF‐1α, and uniformly dispersed into the hydrogel precursor solution to construct the fluidic biomimetic niche (GelSSO/PDA@SDF). Upon minimally invasive injection, GelSSO/PDA@SDF precisely conformed to defect geometry and photocrosslinked in situ into a structural niche seamlessly fused to the surrounding bone interface. In the initial phase of signal amplification, PDA@SDF NPs were preferentially released from the crosslinked network, with SDF‐1α persistently providing chemotactic signals to mobilize endogenous cell homing while synergistically activating vascular niche signals for angiogenesis. Concurrently, PDA NPs altered macrophage phenotype, rectifying local niche immunological imbalance. Subsequently, gradual matrix degradation initiated the secondary signal amplification phase, in which sustained SSO release established a transcriptionally active osteogenic niche, directing continuous osteogenesis of recruited stem cells in coordination with pre‐formed neovasculature. Finally, the efficacy of in situ injected GelSSO/PDA@SDF in promoting vascularized bone regeneration and local immunomodulation was validated in a diabetic rat cranial defect model. Furthermore, this tunable fluidic biomimetic niche holds promise for extension to other poorly regenerative targets, such as the myocardium, central nervous system, and tendons.

## 2 Results and Discussion

### 2.1 Synthesis and Characterization of SDF‐1α‐Loaded PDA NPs

**Figure 1:**
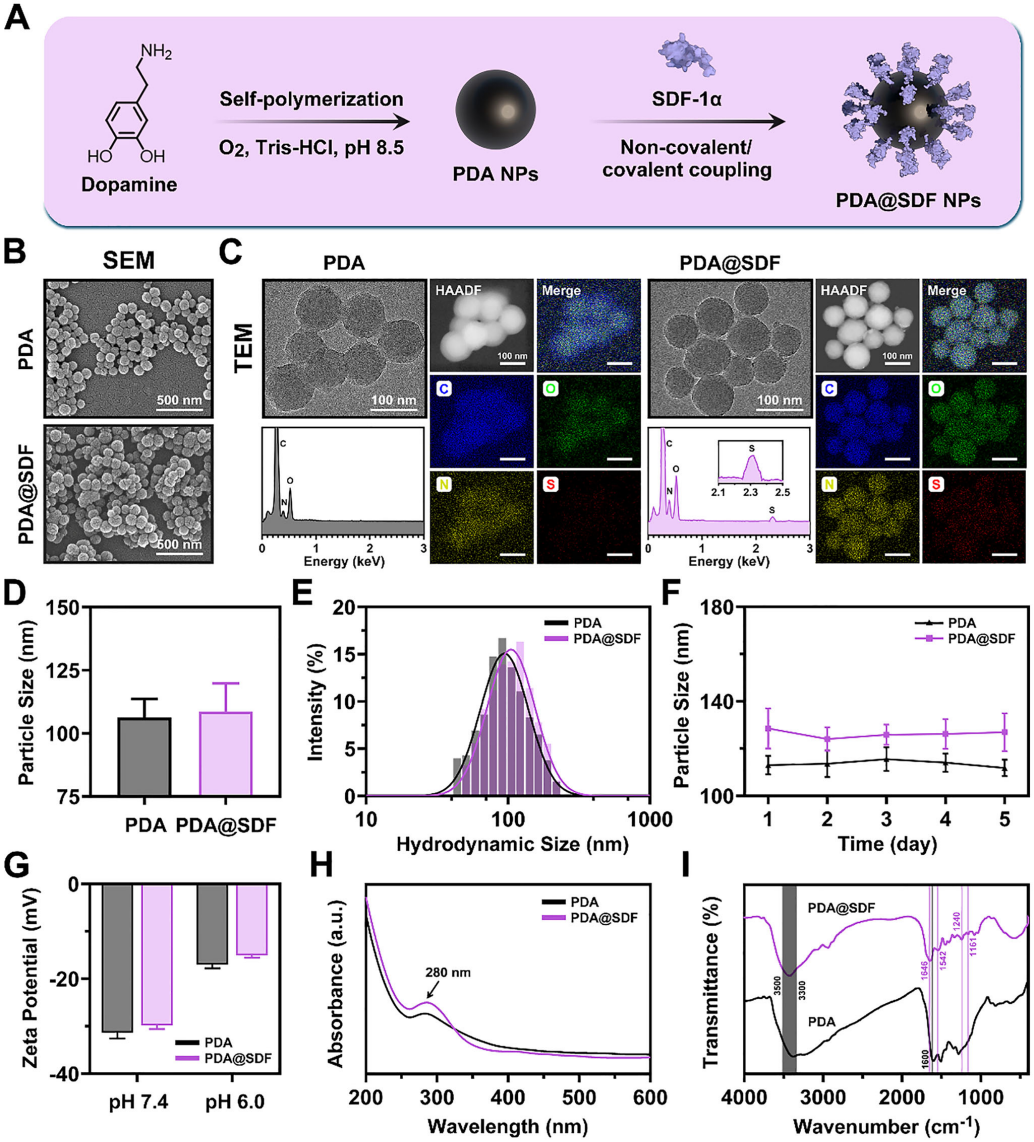
Synthesis and characterization of PDA@SDF NPs. A) Schematic illustration of PDA NP synthesis via dopamine autoxidation and subsequent SDF‐1α loading. B) Representative SEM micrographs of PDA and PDA@SDF NPs. C) Representative TEM images of PDA and PDA@SDF NPs, with corresponding EDS spectra and elemental mapping. D) Average particle size quantification from TEM images (*n* = 100). E) Hydrodynamic sizes and F) dispersion stability of PDA and PDA@SDF NPs in aqueous solution (*n* = 3). G) Zeta potentials of NPs at different pH values (*n* = 3). H) UV–vis and I) FTIR spectra of PDA and PDA@SDF NPs. (Data are presented as mean ± SD).

PDA NPs possess a melanin‐like backbone, which confers intrinsic biocompatibility and a high density of surface‐active sites, rendering them ideal carriers for mild and efficient conjugation of fragile cytokines.^[^ ^41^, ^42^ ^]^ Here, PDA NPs were strategically employed as nanocarriers for SDF‐1α, and PDA@SDF NPs were synthesized via a two‐step method to serve as the “early signal amplification module” of the fluidic biomimetic niche (**Figure** **1**A). Commonly used methods to synthesize PDA NPs include electropolymerization, enzyme‐catalyzed polymerization, and alkaline solution oxidation.^[^ ^43^ ^]^ Due to the operational simplicity and mild reaction conditions, the alkaline oxidation route was selected for nanoparticle synthesis in this study. Under sufficient dissolved oxygen and alkaline pH, dopamine hydrochloride underwent spontaneous oxidative polymerization to yield PDA NPs, accompanied by a visible color change from colorless to deep black as an indicator of polymerization progression (Figure S1A,B, Supporting Information). Purified PDA NPs were subsequently redispersed in an alkaline aqueous medium and complexed with SDF‐1α through reversible covalent and non‐covalent interactions. Finally, PDA@SDF NPs were obtained by freeze‐drying (Figure S2, Supporting Information).

After synthesis, scanning electron microscopy (SEM) revealed that both nanoparticle types were uniformly spherical, without evidence of fusion or flake‐like aggregation, indicating controlled synthesis and a narrow morphological distribution (Figure 1B). Transmission electron microscopy (TEM) further validated their structural integrity and dispersion quality, as reflected by distinct particle boundaries and the absence of internal hollows or structural deformation (Figure 1C). Energy‐dispersive X‐ray spectrometry (EDS) and elemental mapping elucidated the surface chemical composition differences between PDA and PDA@SDF NPs. Pristine PDA NPs exhibited characteristic signatures of carbon (C), oxygen (O), and nitrogen (N), consistent with the polymeric backbone structure.^[^ ^43^ ^]^ In contrast, PDA@SDF NPs displayed an additional sulfur (S) peak near 2.3 keV, attributable to the cysteine residues in SDF‐1α.^[^ ^24^, ^44^ ^]^ The homogeneous distribution of S across mapping images confirmed that SDF‐1α was evenly conjugated onto the nanoparticle surface.

Dynamic light scattering (DLS) analysis revealed that both NPs exhibited single, narrow hydrodynamic size distributions, predominantly within the range of 90–120 nm, which closely matched the TEM‐derived mean diameters of 106.3 ± 2.4 nm for PDA and 108.7 ± 4.7 nm for PDA@SDF (Figure 1D,E). These findings further verified their superior monodispersity and colloidal stability, characteristics that facilitate homogeneous nanoparticle dispersion within the fluidic biomimetic niche, minimize aggregation during injection, and ensure optimal injectability and consistent signal distribution.^[^ ^45^ ^]^ Continuous DLS monitoring over 5 days revealed negligible fluctuations in the hydrodynamic size of PDA and PDA@SDF NPs, suggesting favorable temporal stability conducive to sustained early‐stage signal amplification within biomimetic niches (Figure 1F). Zeta potential measurements confirmed the anticipated shift in nanoparticle surface charge, as shown in Figure 1G. At physiological pH 7.4, PDA NPs exhibited −31.4 ± 1.5 mV, due to the strong negative charge from surface deprotonated phenolic hydroxyl groups. After SDF‐1α loading, the value rose slightly to −29.8 ± 0.7 mV, reflecting partial charge neutralization by cationic residues. As the pH dropped to 6.0, corresponding values shifted further to −17.0 ± 2.0 mV (PDA) and −15.1 ± 0.5 mV (PDA@SDF). Thus, NPs retain sufficient electrostatic repulsion for colloidal stability under mildly acidic inflammatory microenvironment, while acidity‐induced attenuation of charge shielding weakens nanoparticle‐protein interactions, facilitating timely SDF‐1α release and thereby supporting activation of the “signal amplification module” during the early inflammatory phase.^[^ ^46^ ^]^

The optical properties of NPs were examined by spectroscopic analysis. In UV–Vis spectra, PDA NPs displayed a characteristic absorption peak at ≈280 nm, corresponding to *π*–*π* electron transitions within the aromatic domains of the polymeric skeleton.^[^ ^47^ ^]^ When loaded with SDF‐1α, a sharper shoulder peak emerged in this band, attributed to the aromatic amino acid residues in the protein.^[^ ^48^ ^]^ The overall spectral profile remained consistent, indicating that the intrinsic optical properties of the PDA core were preserved (Figure 1H). Fourier transform infrared spectroscopy (FTIR) further showed broad absorption bands at 3300–3500 cm^−1^ for both NPs, assignable to overlapping O─H and N─H stretching vibrations of phenolic and amino groups, respectively. The peak at ≈1600 cm^−1^ originated from C═C stretching of the aromatic ring skeleton.^[^ ^49^ ^]^ In PDA@SDF NPs, new peaks appeared at 1646 and 1542 cm^−1^, corresponding to the amide I (C═O stretching) and amide II (N─H bending and C─N stretching) bands of the protein, respectively.^[^ ^50^ ^]^ Moreover, the enhanced C─N stretching absorption at 1240 cm^−1^ and the altered C─O/C─N vibration region near 1161 cm^−1^ suggested the formation of Schiff base and Michael addition linkages between the quinone groups on PDA and lysine residues of SDF‐1α (Figure 1I).^[^ ^51^ ^]^ Collectively, these spectral features confirm the stable anchoring of SDF‐1α to the nanoparticle surface through a combination of covalent and non‐covalent interactions.

### 2.2 Fabrication and Characterization of GelSSO/PDA@SDF Biomimetic Niche

**Figure 2:**
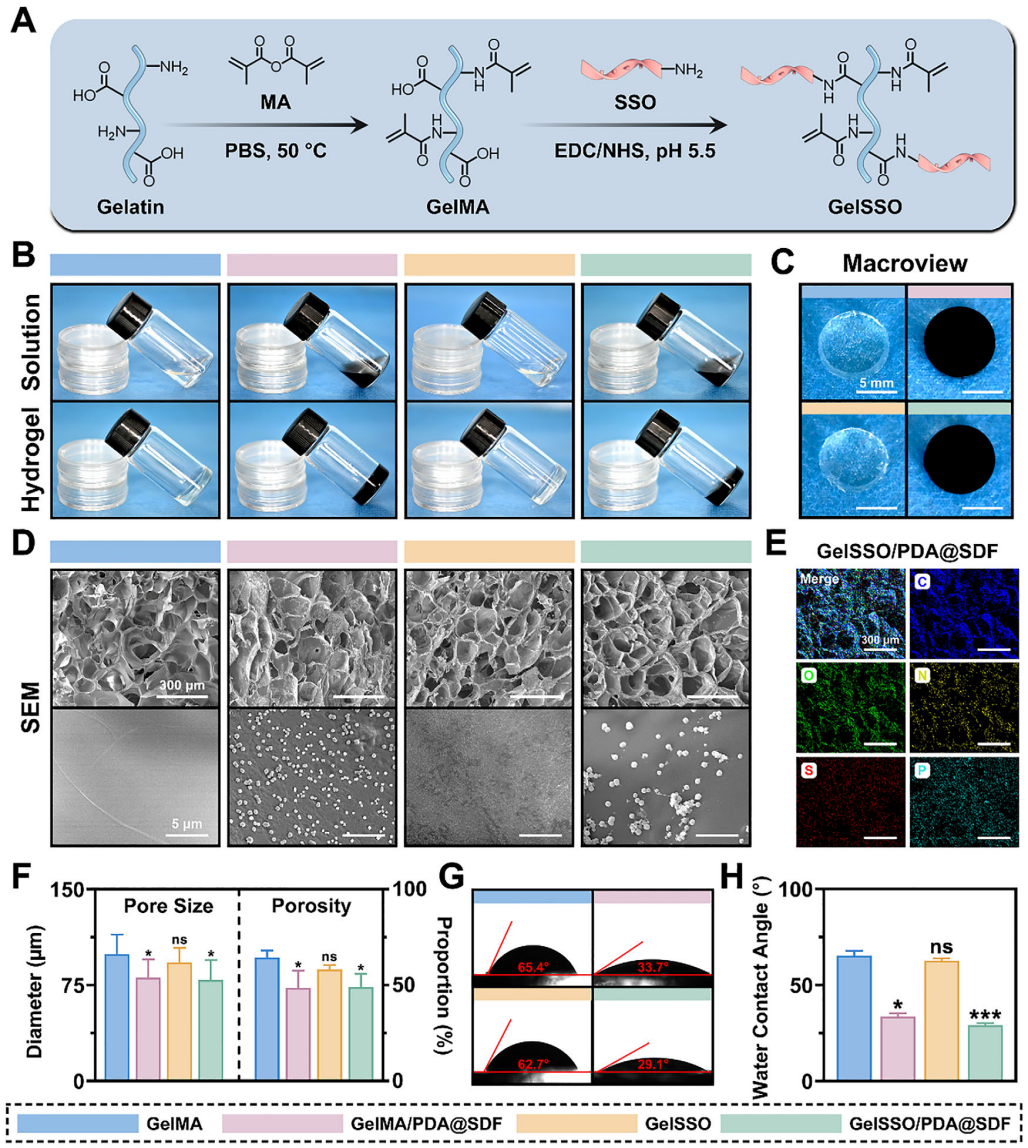
Fabrication and morphological characterization of the biomimetic niche. A) Schematic depicting the sequential chemical modification from gelatin to GelSSO. B) Gelation process of different hydrogels illustrated via tilted‐vial test. C) Macroscopic appearance of hydrogels after photocrosslinking. D) Representative SEM micrographs of various hydrogels, and F) quantitative analysis of pore size and porosity (*n* = 100). E) EDS elemental mapping of GelSSO/PDA@SDF hydrogel. G) Static WCA images and H) quantification for each hydrogel (*n* = 3). (Data are presented as mean ± SD, ns, not significant, *p < 0.05, ***p < 0.001 compared with the GelMA group).

To impart a degradation‐coupled, osteogenic transcription module to the fluidic biomimetic niche, gelatin was modified in two successive steps (**Figure** **2**A). First, methacrylation converted native gelatin into photocurable gelatin methacryloyl (GelMA), allowing the precursor to be injected and rapidly crosslinked in irregular cranial defects. Second, a SSO bearing a 5′‐terminal amine and a nuclease‐resistant phosphorothioate backbone was covalently grafted onto GelMA via 1‐ethyl‐3‐(3‐dimethylaminopropyl) carbodiimide hydrochloride/N‐hydroxysuccinimide (EDC/NHS) chemistry, yielding GelSSO. Because the SSO encodes an osteoinductive motif, its gradual release during matrix degradation provides a second wave of gene regulatory signals for homed stem cells. Finally, PDA@SDF NPs and photoinitiator were uniformly incorporated into the GelSSO precursor, producing an injectable GelSSO/PDA@SDF fluidic biomimetic niche with integrated early and late signal modules.

The ^1^H nuclear magnetic resonance (^1^H NMR) spectra provided direct evidence for successful sequential modification (Figure S3, Supporting Information). GelMA exhibited characteristic vinyl proton doublets at 5.60 and 5.32 ppm, verifying the efficient methacrylation of the gelatin backbone.^[^ ^52^, ^53^ ^]^ In GelSSO, these vinyl signals persisted, while additional aromatic proton peaks (7.83–7.61 ppm) and sugar‐ring resonances (6.10–5.92 ppm) emerged, consistent with the presence of grafted SSO chains.^[^ ^54^ ^]^ Notably, a slight upfield shift in the aromatic region relative to free SSO reflected an altered electronic environment around the newly formed amide bond, corroborating the covalent linkage of SSO onto the GelMA matrix. The tilted‐vial test intuitively demonstrated rapid transition of the precursor solution from a flowable “fluidic niche” into a self‐supporting “structural niche” under visible light irradiation (Figure 2B). Quantitative gelation measurements showed average crosslinking times of 18.6 s for GelMA, 22.8 s for GelMA/PDA@SDF, 20.4 s for GelSSO, and 24.7 s for GelSSO/PDA@SDF (Figure S4, Supporting Information). The modest delay introduced by nanoparticle incorporation and SSO grafting was attributed to partial attenuation of incident light by the PDA melanin‐like framework and a slight increase in viscosity of the precursor.^[^ ^55^, ^56^ ^]^ Nevertheless, the fluidic biomimetic niche solidified well within the clinically acceptable tens‐of‐seconds window, confirming its potential for rapid, controllable photopolymerization and in situ shaping in geometrically complex cranial defects.

Macroscopically, the structured biomimetic niche displayed uniformly black coloration, implying well‐dispersed NPs throughout the hydrogel bulk (Figure 2C). SEM imaging further substantiated this at the microscale, revealing an interconnected porous architecture with homogeneously distributed bright NPs along pore walls and no visible aggregation (Figure 2D). Consistently, EDS elemental mapping showed ubiquitous C, O, and N signals from the gelatin matrix, a clear S signal belonging to SDF‐1α‐loaded PDA NPs, and a homogeneous phosphorus (P) signal from phosphorothioated SSO strands (Figure 2E).^[^ ^24^, ^44^, ^57^, ^58^ ^]^ The simultaneous presence and uniform distribution of S and P verified effective integration of both early (PDA@SDF) and late (SSO) signal amplification modules within the hydrogel network. Crucially, PDA@SDF NPs acted as nanofillers and supplementary physical crosslinking points, tightening the polymer network and modulating the hydrogel architecture.^[^ ^59^ ^]^ As evidenced by quantitative SEM analysis in Figure 2F, the average pore size decreased markedly from 99.3 µm in GelMA to 79.0 µm in GelSSO/PDA@SDF, accompanied by a parallel reduction in overall porosity. This mild densification preserved interconnected channels, which are essential for nutrient convection, staged signal diffusion, and cellular infiltration, while simultaneously endowing the hydrogel with greater mechanical robustness.^[^ ^52^ ^]^ Static water contact angle (WCA) tests found that incorporating PDA@SDF NPs significantly reduced the WCA of the hydrogel compared with SSO grafting alone (Figure 2G,H), attributed to enhanced hydrophilicity originating from the catechol and amine groups on PDA.

**Figure 3:**
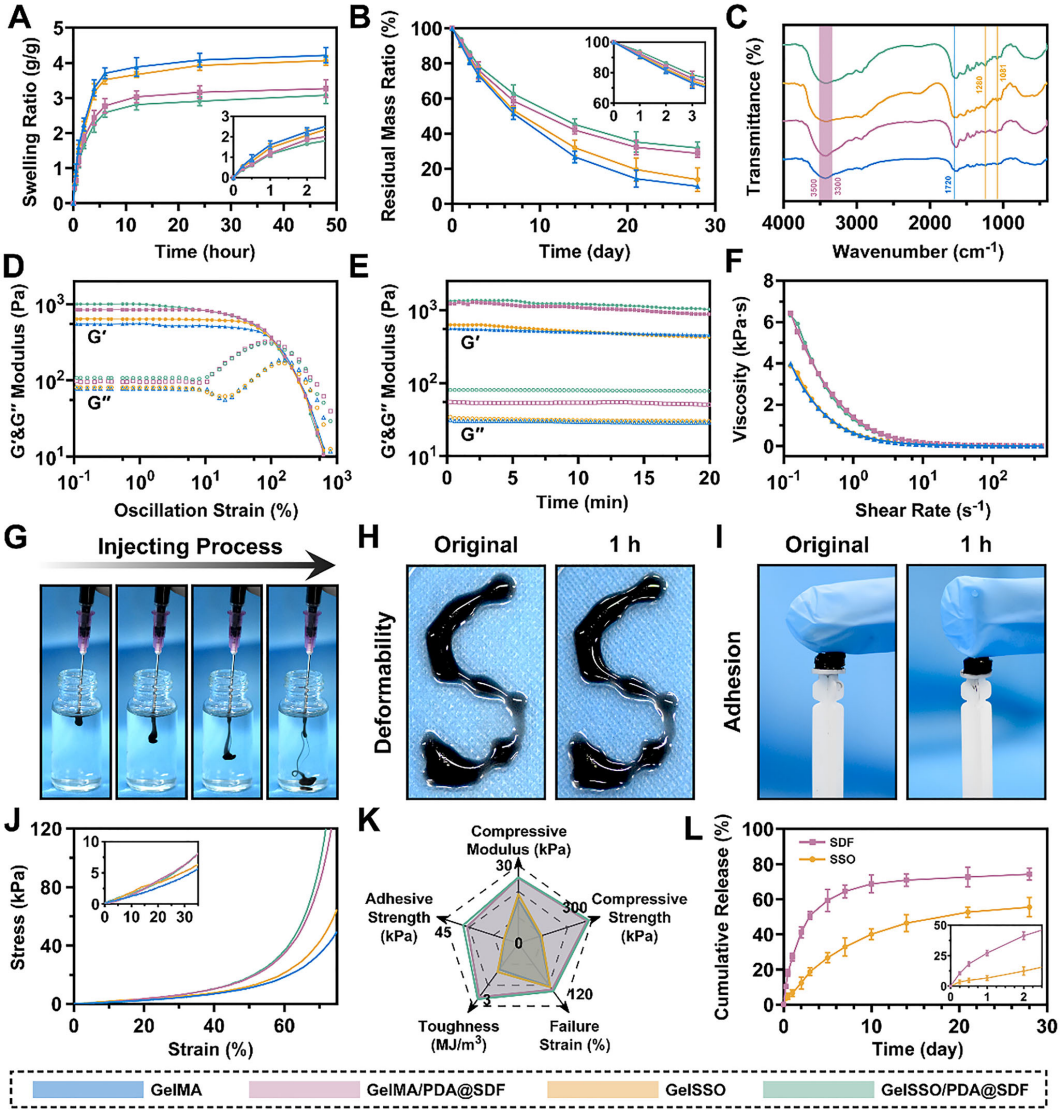
Physicochemical and mechanical characterization of the biomimetic niche. A) Swelling and B) biodegradation behaviors of different hydrogels. C) FTIR spectra of the hydrogels. D) Oscillatory strain and E) time sweep rheological analysis of the hydrogels. F) Shear‐thinning behavior of hydrogel precursors. G) Injectability, H) deformability, and I) adhesion of the GelSSO/PDA@SDF hydrogel. J) Compressive stress–strain curves of various hydrogels. K) Radar chart summarizing mechanical and adhesive parameters of the hydrogels. L) In vitro cumulative release profiles of SDF‐1α and SSO from GelSSO/PDA@SDF hydrogel. (Data are presented as mean ± SD, *n* = 3).

The swelling and biodegradation behaviors of the hydrogels in collagenase‐containing phosphate‐buffered saline (PBS) further confirmed the macroscopic effects of network densification. Native GelMA exhibited the highest water uptake, whereas incorporation of PDA@SDF NPs decreased the equilibrium swelling ratio (ESR) from 4.22 to 3.27 g g^−1^ (**Figure** **3**A; Figure S5, Supporting Information). This compacted mesh likewise retarded hydrolytic and enzymatic erosion. After 28 days of incubation, pristine GelMA retained only 9.97% of its initial mass, whereas GelSSO/PDA@SDF preserved 31.88% (Figure 3B). By limiting water ingress and enzyme diffusion, the densified network prolongs niche geometry and mechanical integrity in vivo, supporting sustained release of both NPs and SSO.^[^ ^60^ ^]^ FTIR spectra further confirmed the sequential construction of the biomimetic niche, as presented in Figure 3C. A prominent band at 1720 cm^−1^ (C═O stretch of methacrylate) verified successful gelatin methacrylation. After SSO grafting, two new signatures appeared at 1280 and 1081 cm^−1^, owing to the asymmetric and symmetric P═O/P─O stretches of the phosphorothioated oligodeoxynucleotide.^[^ ^56^, ^61^ ^]^ The broad hydrogen bond envelope at 3300–3500 cm^−1^, inherent to gelatin, became markedly intensified upon PDA@SDF incorporation, reflecting additional phenolic O─H and protein N─H groups on the NPs.

Rheological tests assessed the mechanical resilience of the structural biomimetic niches and the injectability of their fluidic precursors. Oscillatory strain sweeps showed a broad storage modulus (G′) plateau at low strains, roughly one order of magnitude higher than the loss modulus (G″), confirming an elastically dominated network. Adding PDA@SDF NPs further increased G′, G″, and the G′–G″ crossover strain, indicating enhanced toughness capable of withstanding larger physiological deformations (Figure 3D).^[^ ^52^, ^62^ ^]^ Time sweeps at constant strain displayed stable plateau moduli over 20 min, demonstrating short‐term mechanical stability suitable for in situ retention while retaining compliance for tissue integration (Figure 3E). Shear‐rate sweeps of precursor solutions revealed pronounced shear‐thinning behavior. Although PDA@SDF incorporation slightly raised low‐shear viscosity due to nanoparticle‐induced hydrodynamic drag, viscosity readily fell to 5.75 Pa·s at high shear rates, ensuring smooth extrusion through clinical‐gauge needles suitable for minimally invasive administration (Figure 3F).^[^ ^11^ ^]^ Uniaxial compression confirmed the structural reinforcement imparted by PDA@SDF NPs. GelSSO/PDA@SDF withstood the highest failure load, followed by GelMA/PDA@SDF, whereas GelMA and GelSSO exhibited comparatively greater deformation (Figure 3J). The compressive modulus rose from 16.7 kPa in GelMA to 25.4 kPa in GelSSO/PDA@SDF, and compressive strength nearly tripled from 88 to 286 kPa. Toughness and fracture strain likewise improved, indicating that the NPs act as energy‐dissipating crosslinking nodes within the gelatin network. Lap‐shear tests further showed wet tissue adhesion of 33.6 kPa for GelSSO/PDA@SDF, far exceeding 8.4 kPa of GelMA (Figure 3K). This combined enhancement of stiffness, energy absorption, and interfacial adhesion is critical for resisting micromotion and sustaining in situ signal delivery.^[^ ^60^, ^63^ ^]^ Based on these mechanical findings, the injectability, deformability, and adhesion of the GelSSO/PDA@SDF fluidic biomimetic niche were further evaluated to demonstrate practical applicability. The precursor exhibited excellent injectability, evidenced by its smooth and continuous extrusion into PBS via syringe (Figure 3G). After photopolymerization, the extruded “S” shape maintained geometric integrity for over 1 h, ensuring defect‐specific shaping and positional stability in vivo (Figure 3H). Moreover, catechol‐mediated interfacial bonding enabled robust adhesion of the cured hydrogel to diverse substrates, supporting suspended loads and confirming durable affinity with moist cranial bone and dura mater (Figure 3I).^[^ ^41^, ^43^ ^]^ Altogether, the combination of smooth injectability, shape fidelity, and durable adhesion satisfies key practical requirements for in situ cranial defect repair.

The cumulative release profiles validated the chronotaxic signal amplification strategy of the biomimetic niche. SDF‐1α in PDA@SDF NPs showed a burst release of 59.3% within the initial 5 days. Under the mildly acidic inflammatory conditions, the reversible Schiff base and Michael addition linkages on PDA are cleaved, while the highly hydrated matrix shortened diffusion pathways, collectively driving this early‐phase signal amplification.^[^ ^41^, ^63^ ^]^ The resulting gradient rapidly recruited endogenous stem cells and endothelial progenitors, simultaneously correcting diabetic inflammation. Release subsequently decelerated, reaching 70.9% by day 14, thereby sustaining angiogenic stimulation via continuous activation of the vascular niche. In contrast, SSO release was slow and sustained, with 18.8% released by day 3 and 55.6% over 28 days. This delayed release was due to the covalent anchoring of SSO onto GelMA, necessitating enzymatic matrix degradation.^[^ ^52^ ^]^ Consequently, this late‐phase signal module provided a prolonged transcriptionally active osteogenic niche, directing stem cell differentiation once vascularization and immune balance were in place (Figure 3L).

### 2.3 In Vitro Biocompatibility of GelSSO/PDA@SDF Biomimetic Niche

Before employing a biomimetic niche for in situ tissue regeneration, it is crucial to verify cytocompatibility comparable to native SCNs, ensuring effective cell survival, adhesion, and proliferation without cytotoxicity.^[^ ^52^, ^59^ ^]^ To determine whether the GelSSO/PDA@SDF biomimetic niche fulfills this prerequisite, we investigated its interactions with two in vitro surrogate cell models representing the target populations expected to be recruited in vivo, namely human umbilical vein endothelial cells (HUVECs) for EPCs and rat bone marrow mesenchymal stem cells (rBMSCs) for BMSCs.

**Figure 4:**
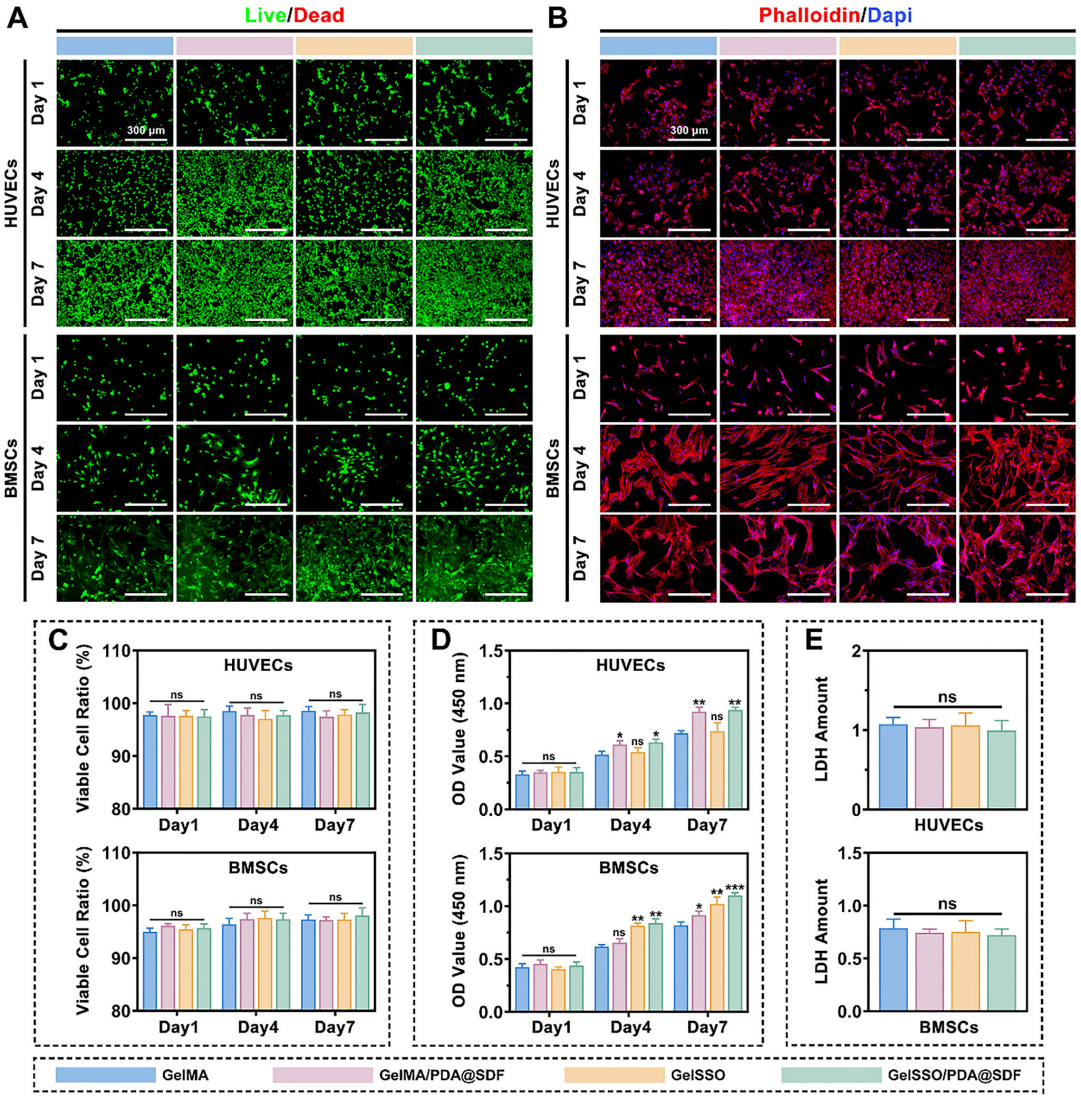
In vitro biocompatibility evaluation of the biomimetic niche. A,B) Representative live/dead and cytoskeletal staining of HUVECs and rBMSCs after hydrogel treatment for 1, 4, and 7 days. C) Quantitative analysis of cell viability based on live/dead staining. D) Cell proliferation of HUVECs and rBMSCs assessed by CCK‐8 assay over 1, 4, and 7 days post‐treatment. E) LDH release from HUVECs and rBMSCs at day 1 post‐treatment, indicating cytotoxicity. (Data are presented as mean ± SD, *n* = 3, ns, not significant, *p < 0.05, **p < 0.01, ***p < 0.001 compared with the GelMA group).

Live/dead staining revealed predominant green fluorescence with negligible red signals in every hydrogel group, accompanied by an increase in cell density from day 1 to 7 (**Figure** **4**A). Quantitative analysis consistently confirmed the high viability of both HUVECs and rBMSCs, without statistically significant differences among groups, demonstrating that neither PDA@SDF incorporation nor SSO grafting induced acute cytotoxicity (Figure 4C). Cytoskeletal staining further confirmed the integrity of the actin architecture in both cell types across all hydrogel groups. Specifically, HUVECs maintained their characteristic cobblestone morphology, indicative of a preserved endothelial phenotype and stable intercellular junctions, without signs of a fibrotic transition.^[^ ^64^, ^65^ ^]^ Conversely, rBMSCs progressively assumed an elongated, spindle‐shaped form on hydrogel surfaces, implying that the peptide‐rich GelMA and hydrophilic PDA components facilitated enhanced adhesion and spreading.^[^ ^41^, ^52^ ^]^ Such enhanced cytoskeletal tension provided a favorable mechanotransductive platform for subsequent osteogenic differentiation (Figure 4B).^[^ ^18^ ^]^ Cell proliferation was quantitatively assessed via the cell counting kit‐8 (CCK‐8) assay, with results presented in Figure 4D. Optical density (OD) values for both HUVECs and rBMSCs gradually increased over 7 days in all hydrogel groups. Compared to GelMA alone, GelMA/PDA@SDF significantly enhanced the growth of both cell lines, whereas GelSSO selectively boosted rBMSCs. These effects were attributable to sustained mitogenic cues derived from released SDF‐1α and the SSO, aligning with previous reports.^[^ ^20^, ^24^, ^66^, ^67^ ^]^ Notably, rBMSCs cultured on GelSSO/PDA@SDF exhibited the highest OD value on day 7, indicating synergistically enhanced proliferative activity in the presence of both PDA@SDF NPs and SSO. Lastly, lactate dehydrogenase (LDH) release was statistically indistinguishable among groups for either HUVECs or rBMSCs, confirming that soluble products from the hydrogels do not compromise membrane stability (Figure 4E). Taken together, these results verify excellent intrinsic cytocompatibility of the GelSSO/PDA@SDF biomimetic niche.

### 2.4 In Vitro Immunomodulatory Activity of GelSSO/PDA@SDF Biomimetic Niche

Acute inflammation is indispensable for debris clearance after injury. However, the diabetic microenvironment often stalls inflammation at the M1 phase, triggering a persistent cytokine burden that hinders cell recruitment, impedes endothelial sprouting, and ultimately impairs bone regeneration.^[^ ^25^, ^68^ ^]^ Accordingly, a biomimetic niche intended for diabetic bone healing must first restore the physiological M1‐to‐M2 macrophage transition to reestablish immune homeostasis. RAW264.7 cells, a murine macrophage line commonly used to study polarization dynamics, reliably adopt an M1‐like phenotype upon stimulation, thereby mimicking the pro‐inflammatory state observed in vivo.^[^ ^69^ ^]^ Here, we pre‐polarized RAW264.7 cells toward an M1‐like phenotype and subsequently assessed whether the GelSSO/PDA@SDF biomimetic niche could redirect them toward an M2‐favored profile, thereby evaluating its immunomodulatory capacity.

**Figure 5:**
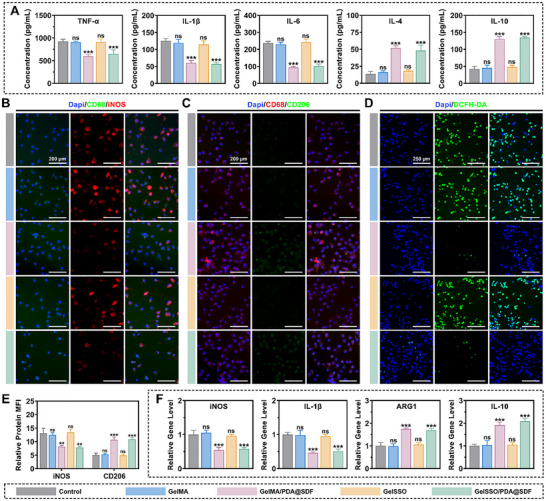
In vitro immunomodulatory potential of the biomimetic niche. A) ELISA quantification of pro‐inflammatory (TNF‐α, IL‐1β, IL‐6) and anti‐inflammatory cytokines (IL‐4, IL‐10) secreted by RAW264.7 macrophages. B,C) Representative immunofluorescence staining of the M1 marker iNOS and the M2 marker CD206. D) Representative immunofluorescence images of intracellular ROS detected by DCFH‐DA. E) Semi‐quantification of relative mean fluorescence intensity (MFI) for iNOS and CD206. F) qRT‐PCR analysis of macrophage polarization‐related genes, including iNOS, IL‐1β, ARG1, and IL‐10. (Data are presented as mean ± SD, *n* = 3, ns, not significant, **p < 0.01, ***p < 0.001 compared with the GelMA group).

To capture the earliest functional shift post‐treatment, we first assessed macrophage cytokine secretion profiles by enzyme‐linked immunosorbent assay (ELISA). As shown in **Figure** **5**A, RAW264.7 cells primed with lipopolysaccharide (LPS) and interferon‐γ (IFN‐γ) in the GelMA group maintained an M1‐like secretory phenotype similar to the induced control, characterized by abundant secretion of pro‐inflammatory mediators including tumor necrosis factor‐α (TNF‐α), interleukin‐1β (IL‐1β), and interleukin‐6 (IL‐6), alongside minimal production of anti‐inflammatory cytokines interleukin‐4 (IL‐4) and interleukin‐10 (IL‐10). In contrast, nanoparticle‐containing hydrogels significantly reduced the secretion of all three pro‐inflammatory cytokines and increased the levels of IL‐4 and IL‐10, whereas grafting SSO alone had a minimal effect. These results indicated PDA@SDF NPs as the primary anti‐inflammatory driver promoting M2‐type repolarization. Next, immunofluorescence staining was performed to verify that these functional changes reflected genuine intracellular reprogramming of macrophages. Cluster of differentiation 68 (CD68) served as a pan‐macrophage marker to ensure that polarization signals originated specifically from macrophages, while inducible nitric oxide synthase (iNOS) and cluster of differentiation 206 (CD206) distinguished M1 and M2 phenotypes, respectively.^[^ ^26^, ^27^, ^69^ ^]^ Compared to the induced control, iNOS remained highly expressed in GelMA and GelSSO groups but was significantly attenuated in hydrogels containing PDA@SDF, whereas CD206 fluorescence was notably enhanced (Figure 5B,C). Semi‐quantitative analysis of mean fluorescence intensity confirmed the nanoparticle‐mediated shift from an M1 to an M2 phenotype (Figure 5E). Next, intracellular oxidative stress was evaluated using the reactive oxygen species (ROS)‐sensitive probe 2′,7′‐dichlorodihydrofluorescein diacetate (DCFH‐DA). GelMA and GelSSO groups exhibited persistent ROS accumulation similar to the induced control. In PDA@SDF‐containing hydrogels, however, ROS fluorescence was markedly reduced (Figure 5D; Figure S6, Supporting Information). Finally, quantitative real‐time PCR (qRT‐PCR) was conducted to validate these shifts at the transcriptional level, showing pronounced suppression of iNOS and IL‐1β mRNA expression, as well as concomitant upregulation of arginase‐1 (ARG1) and IL‐10, specifically in the GelMA/PDA@SDF and GelSSO/PDA@SDF groups compared with the induced control (Figure 5F). Mechanistically, PDA NPs scavenge ROS, attenuate nuclear factor‐kappa B pathway activation, and consequently reduce downstream iNOS and IL‐1β production. Meanwhile, their surface catechol groups further facilitate M2 polarization by activating signal transducer and activator of transcription 6.^[^ ^70^, ^71^, ^72^, ^73^ ^]^ Although SSO release does not directly influence inflammation, it contributes to overall balanced immunoregulation. Collectively, the GelSSO/PDA@SDF biomimetic niche reverses the pathological M1 polarization characteristic of diabetic defects, facilitates inflammation resolution, and establishes a M2‐dominated niche conducive to subsequent regenerative events.

### 2.5 In Vitro Chemotactic Capacity of GelSSO/PDA@SDF Biomimetic Niche

Cranial bone healing hinges on the timely influx of EPCs and BMSCs. Yet, its native niche is sparse and fails to provide substantial cellular reservoirs or sufficiently potent chemotactic signals for homing. To address this limitation, the GelSSO/PDA@SDF biomimetic niche was designed to augment endogenous cell recruitment. Therefore, its chemotactic performance toward these critical reparative populations was systematically evaluated in vitro, alongside the underlying mechanism.

**Figure 6:**
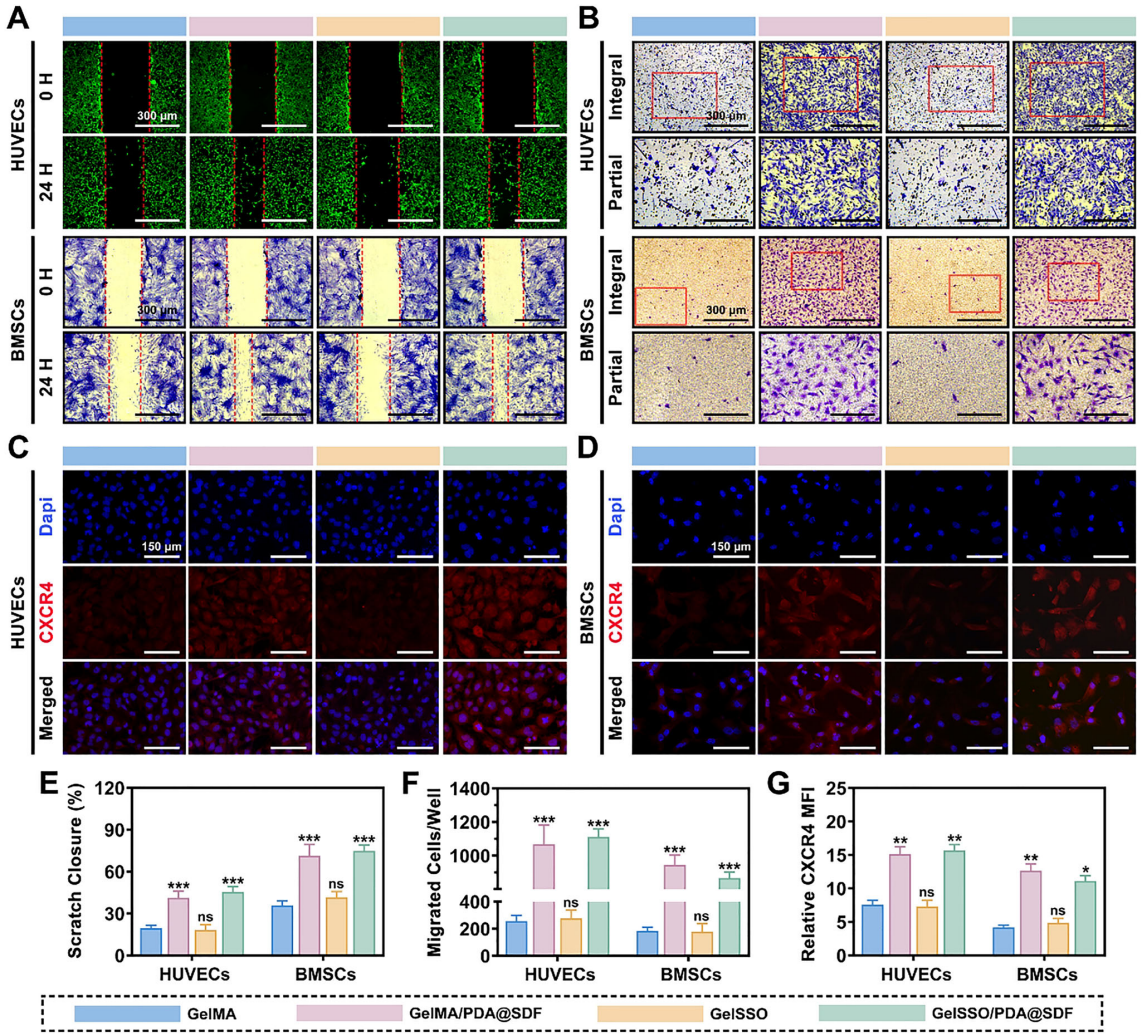
In vitro chemotactic potential of the biomimetic niche. A) Representative wound healing images of HUVECs and rBMSCs at 0 and 24 h, with E) quantification of scratch closure rates. B) Representative images of crystal violet‐stained migrated HUVECs and rBMSCs after 24 h, with F) quantification of migrated cells. C,D) Representative immunofluorescence staining of the chemokine receptor CXCR4 in migrated HUVECs and rBMSCs. G) Semi‐quantification of relative mean fluorescence intensity (MFI) for CXCR4. (Data are presented as mean ± SD, *n* = 3, ns, not significant, *p < 0.05, **p < 0.01, ***p < 0.001 compared with the GelMA group).

Wound healing and Transwell migration assays were conducted to mimic the 2D surface diffusion and 3D matrix invasion that occur during defect repair. In the wound healing assay, both HUVECs and BMSCs exhibited comparable closure rates in the GelMA and GelSSO groups after 24 h, whereas hydrogels containing PDA@SDF NPs significantly accelerated gap closure (**Figure** **6**A,E). These findings suggest that the NPs enhance lateral cell migration, enabling the rapid formation of a cellular sheet along the exposed edges to initiate surface sealing and prime subsequent tissue infill. Consistent trends were observed in Transwell migration assays, wherein the number of migrated HUVECs and BMSCs significantly increased in the GelMA/PDA@SDF and GelSSO/PDA@SDF groups relative to the GelMA group, whereas the GelSSO group showed no marked improvement (Figure 6B,F). These results highlighted the role of NPs in promoting 3D infiltration into deeper defect sites, crucial for adequately filling defect volumes with nascent vasculature and osteogenic tissue. To elucidate the potential recruitment mechanism, immunofluorescence staining was performed on migrated cells. Results revealed that the fluorescence intensity of CXC chemokine receptor type 4 (CXCR4) expressed on both HUVECs and BMSCs was notably elevated in nanoparticle‐containing hydrogel groups (Figure 6C,D,G). It is well established that SDF‐1α binds specifically to its receptor, CXCR4, forming the SDF‐1α/CXCR4 axis, which critically regulates directional cell migration toward injured tissues.^[^ ^16^, ^22^, ^24^ ^]^ By amplifying this signaling axis via sustained release of abundant SDF‐1α, CXCR4‐positive endothelial and mesenchymal progenitor cells were continuously aggregated into the defect area.^[^ ^20^ ^]^ Therefore, the biomimetic niche activated CXCR4 receptors on distant BMSCs and HUVECs through controlled SDF‐1α delivery, establishing a stable chemotactic signal and thereby driving sustained, directed cellular migration via the SDF‐1α/CXCR4 signaling axis.

### 2.6 In Vitro Angio‐Osteogenic Potential of GelSSO/PDA@SDF Biomimetic Niche

Having demonstrated that the GelSSO/PDA@SDF biomimetic niche mitigates excessive inflammation and reinforces endogenous cell recruitment, the subsequent challenge involves sustaining the maturation of vasculature and mineralized matrix, both critical determinants for effective long‐term cranial bone repair.^[^ ^7^, ^17^ ^]^ These interconnected processes are intrinsically slow within the calvarium, due to the scarcity of active vascular and osteogenic niches, becoming even more quiescent after injury. For this, the present biomimetic niche was engineered to achieve the sustained release of SDF‐1α, thereby maintaining a pro‐angiogenic vascular niche, while continuously liberating SSO, which delivers a genetic osteoinductive signal to establish a transcriptionally active osteogenic niche. Here, we systematically validated the capability of the GelSSO/PDA@SDF biomimetic niche to promote angiogenesis and osteogenesis in vitro, including elucidation of the underlying signaling mechanisms.

**Figure 7:**
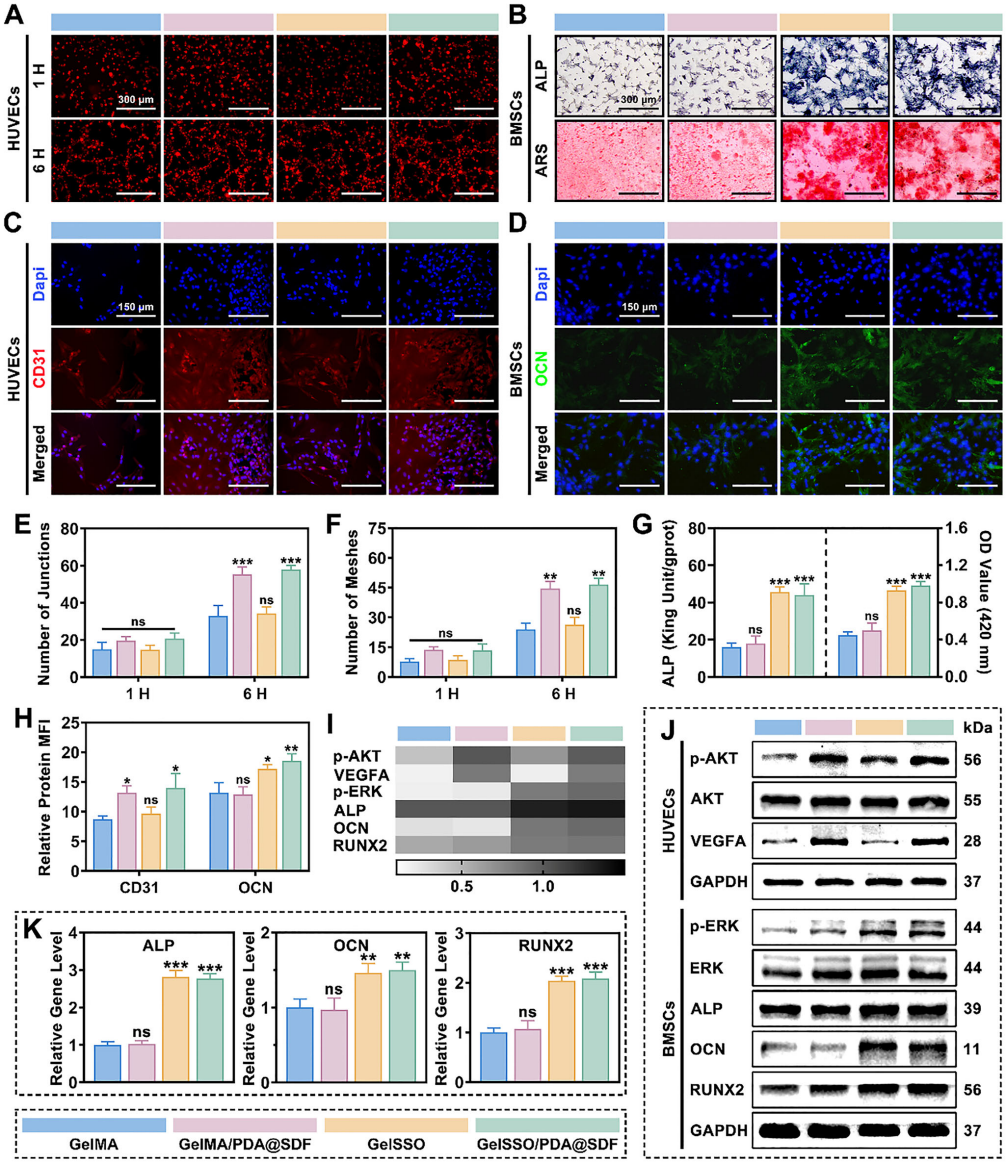
In vitro angiogenic and osteogenic potential of the biomimetic niche. A) Representative images of HUVEC tube formation at 1 and 6 h, and E,F) quantitative analysis of junction and mesh numbers. B) Representative ALP staining (day 7) and ARS staining (day 21) of rBMSCs, along with G) corresponding quantitative analyses of osteogenic differentiation. C,D) Representative immunofluorescence staining of the endothelial marker CD31 (day 3) and the osteogenic marker OCN (day 21). H) Semi‐quantification of relative mean fluorescence intensity (MFI) for CD31 and OCN. I,J) Western blotting and corresponding densitometric heatmap analyses of angiogenesis‐related proteins (VEGFA, AKT, p‐AKT, day 3) and osteogenesis‐related proteins (ALP, OCN, RUNX2, ERK, p‐ERK, day 7). K) qRT‐PCR analysis of osteogenic gene expression (ALP, OCN, RUNX2) on day 7. (Data are presented as mean ± SD, *n* = 3, ns, not significant, *p < 0.05, **p < 0.01, ***p < 0.001 compared with the GelMA group).

Initially, the angio‐osteogenic potential was evaluated at the morphological level. In tube formation assays, HUVECs seeded on Matrigel began assembling into capillary‐like networks within the first hour across all hydrogel groups (**Figure** **7**A). Although junction and mesh counts showed no significant differences at this early stage, both nanoparticle‐containing groups (GelMA/PDA@SDF and GelSSO/PDA@SDF) displayed a discernible upward trend, suggesting accelerated network initiation (Figure 7E,F). By 6 h, these initial advantages translated into denser networks, characterized by significantly higher junction and mesh counts compared to the GelMA group. These observations indicated that nanoparticle‐released SDF‐1α accelerates endothelial morphogenesis, promoting the organization of endothelial cells into more mature vascular structures over time. Alkaline phosphatase (ALP) activity, indicative of early osteogenic commitment, and calcium nodule deposition, representative of mature matrix mineralization, were assessed in rBMSCs cultured on various hydrogels at day 7 and 21, respectively. ALP staining on day 7 revealed sparse, pale precipitates in GelMA and GelMA/PDA@SDF groups, whereas dense, blue‐violet deposits extensively covered cultures with SSO‐grafted hydrogels. Correspondingly, by day 21, Alizarin Red S (ARS) staining showed abundant deep‐red mineralized nodules exclusively in GelSSO and GelSSO/PDA@SDF groups, contrasting with scant mineralization in other groups (Figure 7B). Quantitative analyses confirmed visual observations, revealing significantly elevated ALP activity and ARS absorbance in GelSSO and GelSSO/PDA@SDF groups relative to GelMA, with negligible differences between GelMA and GelMA/PDA@SDF (Figure 7G). Thus, gradual GelSSO degradation provides sustained SSO release, accelerating both initiation and maturation phases of osteogenesis in rBMSCs. Immunofluorescence staining further supported these morphological findings. Expression of cluster of differentiation 31 (CD31), a junctional protein indicative of endothelial maturation, exhibited notably brighter red fluorescence in HUVECs cultured on nanoparticle‐containing hydrogels compared to those cultured on GelMA alone (Figure 7C).^[^ ^53^ ^]^ Similarly, osteocalcin (OCN), a marker of late‐stage bone matrix maturation, displayed significantly stronger green fluorescence signals in rBMSCs cultured on SSO‐grafted hydrogels (Figure 7D).^[^ ^20^ ^]^ Semi‐quantitative fluorescence intensity analysis reinforced these trends, confirming significant enhancements in CD31 and OCN expression within respective hydrogel groups (Figure 7H). Next, osteogenic gene expression in rBMSCs was quantified via qRT‐PCR to assess osteoinductive effects at the transcriptional level. Expression levels of ALP, OCN, and runt‐related transcription factor 2 (RUNX2) were significantly upregulated in both SSO‐grafted groups compared to the GelMA group (Figure 7K).

Following morphological and molecular characterizations, we investigated the intracellular signaling pathways underpinning the angiogenic and osteogenic activities induced by the GelSSO/PDA@SDF biomimetic niche. Previous studies have established that endothelial cell migration and angiogenic sprouting are primarily mediated by the phosphoinositide 3‐kinase (PI3K)/protein kinase B (AKT) signaling pathway. In contrast, osteogenic differentiation is predominantly regulated by the mitogen‐activated protein kinase (MAPK)/extracellular signal‐regulated kinase (ERK) signaling.^[^ ^20^, ^24^, ^74^, ^75^, ^76^ ^]^ Accordingly, we examined the expression levels of phosphorylated AKT (p‐AKT) and vascular endothelial growth factor A (VEGFA) in HUVECs, and assessed phosphorylated ERK (p‐ERK), RUNX2, ALP, and OCN in rBMSCs. Western blot analysis revealed markedly elevated phosphorylation of AKT and increased levels of its downstream effector, VEGFA, in nanoparticle‐containing hydrogels, indicating enhanced endothelial activation via the sustained release of SDF‐1α, which activates the AKT pathway. Concurrently, SSO‐grafted hydrogels significantly increased ERK phosphorylation, RUNX2 expression, and protein levels of ALP and OCN in rBMSCs, suggesting that SSO‐triggered MAPK/ERK activation promotes osteogenic differentiation and matrix maturation (Figure 7J). Band intensity heatmap analysis further substantiated these trends, confirming that the biomimetic niche potentiates angiogenesis through SDF‐1α‐driven AKT signaling and promotes osteogenesis via SSO‐induced MAPK/ERK activation (Figure 7I).

### 2.7 GelSSO/PDA@SDF Biomimetic Niche Promotes Vascularized Cranial Bone Regeneration in a Diabetic Rat Model

**Figure 8:**
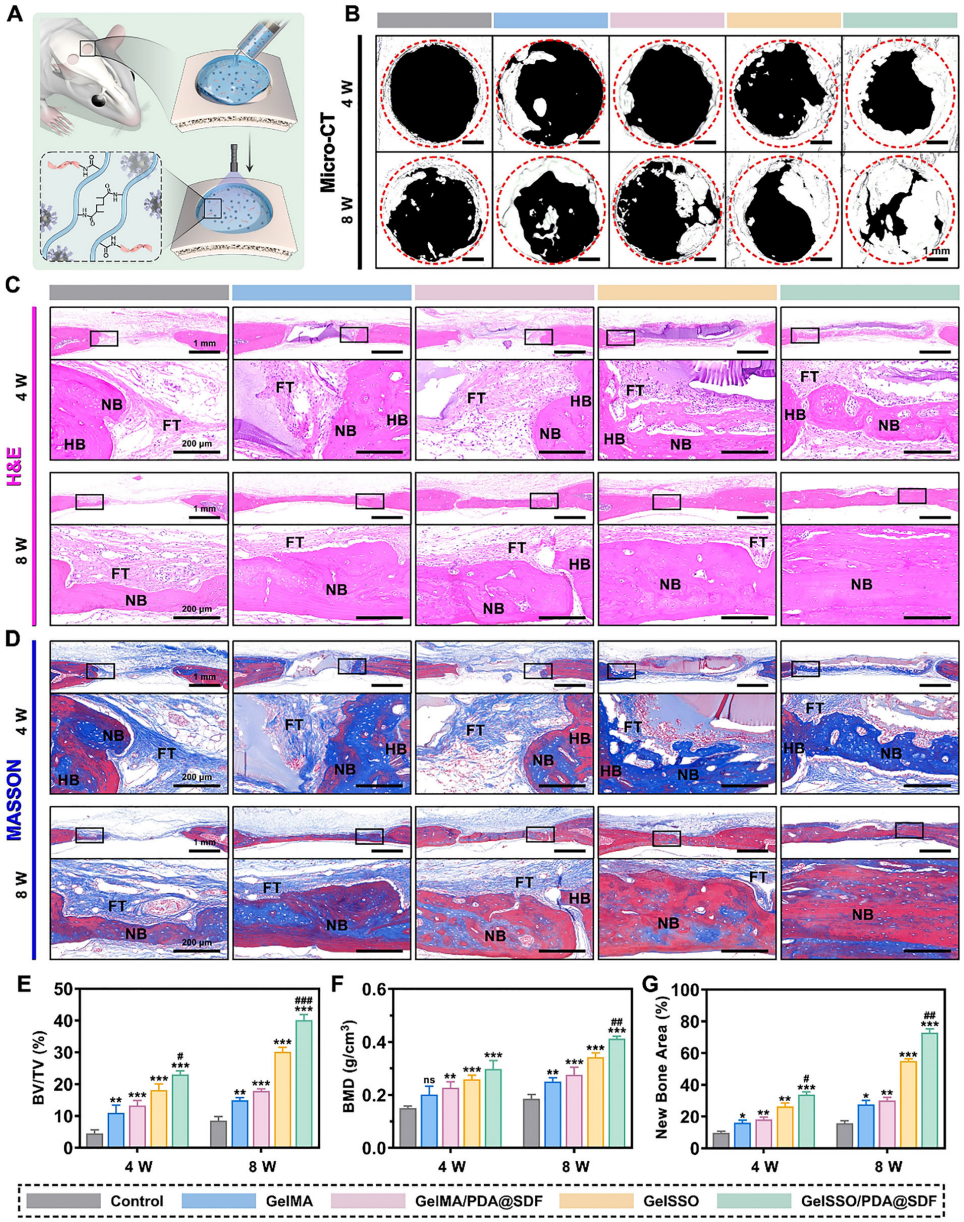
In vivo evaluation of bone regeneration in diabetic cranial defects after biomimetic niche implantation. A) Schematic illustration of in situ injection and crosslinking of the fluidic biomimetic niche. B) Representative 3D micro‐CT reconstructions at 4 and 8 weeks post‐implantation. Red dashed circles delineate original defect boundaries. C,D) Representative H&E and Masson's trichrome staining at 4 and 8 weeks post‐implantation, with HB, NB, and FT indicating host bone, new bone, and fibrous tissue, respectively. E,F) Quantitative analysis of BV/TV and BMD within defect areas. G) Quantification of the new bone area proportion. (Data are presented as mean ± SD, *n* = 4, ns, not significant, *p < 0.05, **p < 0.01, ***p < 0.001 compared with the control group, # *p* < 0.05, ## *p* < 0.01, ### *p* < 0.001 compared with the GelSSO group).

Given the proven stage‐specific signal amplification of GelSSO/PDA@SDF for immunomodulation, cell recruitment, and angio‐osteogenesis in vitro, a streptozotocin (STZ)‐induced diabetic rat cranial defect model was employed to further validate its chronotaxic signal amplification behavior in vivo.^[^ ^68^ ^]^ Following the creation of bilateral full‐thickness defects in the parietal bone, the injectable fluidic biomimetic niche was locally delivered and photopolymerized to form a conformal structural niche (**Figure** **8**A; Figure S7, Supporting Information). At 1 week post‐implantation, immunofluorescence staining was conducted to assess early immune response and progenitor cell recruitment. As shown in Figure S8A (Supporting Information), strong iNOS and weak CD206 fluorescence were observed in the control, GelMA, and GelSSO groups, indicating persistent M1 macrophage dominance in the diabetic defect. Conversely, both the GelMA/PDA@SDF and GelSSO/PDA@SDF groups exhibited a marked reduction in iNOS and an enrichment of the CD206 signal, indicating a shift toward an M2 reparative phenotype. Semi‐quantification of mean fluorescence intensity (MFI) confirmed significant suppression of iNOS and enhancement of CD206 exclusively in the nanoparticle‐containing hydrogels (Figure S8B, Supporting Information). Moreover, immunostaining for cluster of differentiation 34 (CD34) and cluster of differentiation 90 (CD90) revealed substantial recruitment of EPCs and mesenchymal stem cells (MSCs) in the PDA@SDF‐containing groups, characterized by extensive infiltration and increased fluorescence signals, while other groups showed sparse staining and low MFI values (Figure S9A–D, Supporting Information).^[^ ^53^, ^74^ ^]^ Altogether, the sustained release of PDA@SDF NPs from the biomimetic niche effectively recalibrated the diabetic inflammatory environment, creating an immunologically favorable niche enriched with recruited progenitor cells for subsequent bone repair.

Radiological and histological evaluations at 4 and 8 weeks demonstrated the long‐term regenerative efficacy of the biomimetic niche. Representative 3D micro‐computed tomography (micro‐CT) reconstructions revealed minimal ossification in untreated defects, which remained largely radiolucent. GelMA alone induced partial infill yet left sizable voids, whereas hydrogels incorporating a single signal amplification module (GelMA/PDA@SDF or GelSSO) generated notable intradefect bone islands. Remarkably, the dual‐module GelSSO/PDA@SDF hydrogel produced the most widespread and continuous bony regeneration, nearly closing the defect by week 8 (Figure 8B). Quantitative analysis corroborated the imaging data, showing that bone volume fraction (BV/TV) and bone mineral density (BMD) increased significantly in all hydrogel groups compared to the control at both time points, with GelSSO/PDA@SDF outperforming every comparator (Figure 8E,F). Histological assessments further supported the radiological findings. Hematoxylin and eosin (H&E) staining demonstrated that at week 4, defects in the control group were predominantly occupied by fibrous tissue, whereas other groups exhibited early woven bone intertwined with degrading hydrogel. By week 8, although all groups showed progressive increases in bone regeneration, the control group remained limited to marginal bone ingrowth. In contrast, all hydrogel groups exhibited complete material resorption, accompanied by partial to substantial formation of bony bridges. Notably, the GelSSO/PDA@SDF group exhibited the most significant bone mass, characterized by continuous lamellar structures indicative of advanced remodeling (Figure 8C,G).^[^ ^56^ ^]^ Masson's trichrome staining similarly illustrated a dense network of disorganized collagen in control defects at both time points, confirming fibrotic healing caused by diabetes. Hydrogel‐treated defects displayed blue‐stained immature bone at week 4, with the GelSSO/PDA@SDF group already forming extensive, contiguous patches of bone. By week 8, collagen deposition and red mineralized matrix had increased across all hydrogel groups. Strikingly, only the GelSSO/PDA@SDF hydrogel fully bridged the defect with densely mineralized, mature lamellar bone, underscoring its superior matrix maturation and structural integrity (Figure 8D).

**Figure 9:**
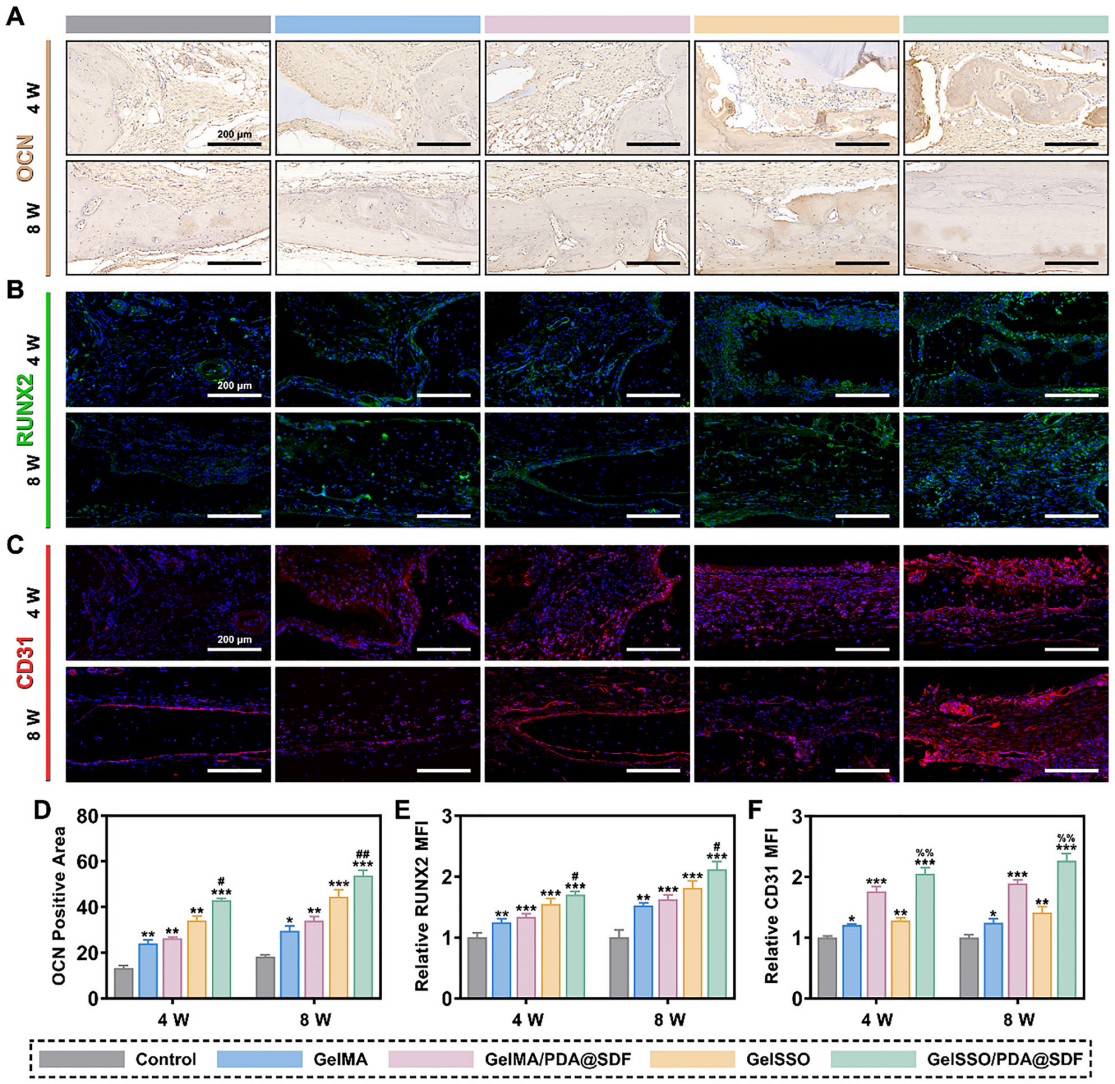
In vivo immunohistological evaluation of bone regeneration in diabetic cranial defects after biomimetic niche implantation. A) Representative immunohistochemical staining and D) semi‐quantitative analysis of the osteogenic marker OCN at 4 and 8 weeks post‐implantation. B, C) Representative immunofluorescence staining of the osteogenic transcription factor RUNX2 and the endothelial marker CD31 at 4 and 8 weeks post‐implantation. E,F) Semi‐quantification of relative mean fluorescence intensity (MFI) for RUNX2 and CD31. (Data are presented as mean ± SD, *n* = 4, ns, not significant, *p < 0.05, **p < 0.01, ***p < 0.001 compared with the control group, %% *p* < 0.01 compared with the GelMA/PDA@SDF group, # *p* < 0.05, ## *p* < 0.01 compared with the GelSSO group).

To elucidate the niche‐mediated regenerative mechanisms, immunohistological analyses of osteogenic and angiogenic markers were conducted. OCN immunohistochemistry revealed progressive matrix protein deposition across all groups, with hydrogel treatments markedly exceeding the control. Among these, GelSSO/PDA@SDF presented the broadest and most intense OCN positivity at both 4 and 8 weeks (**Figure** **9**A,D). Consistent patterns were observed in immunofluorescence staining of RUNX2 and CD31. At both time points, the GelSSO/PDA@SDF hydrogel consistently induced the highest RUNX2 signal and the most excellent density of CD31‐positive microvessels, surpassing hydrogels containing a single signal module in promoting osteogenesis and neovascularization (Figure 9B,C,E,F).

Overall, through the sequential and sustained release of PDA@SDF NPs and SSO, the biomimetic niche promoted coupled angiogenesis and osteogenesis, ultimately achieving vascularized bone regeneration in diabetic defects.

Furthermore, systemic biosafety was verified by histology and serum biochemistry at 8 weeks post‐implantation. H&E staining of major organs displayed intact architecture without inflammation, necrosis, or fibrosis across all groups (Figure S10, Supporting Information). Serum alanine aminotransferase (ALT), aspartate aminotransferase (AST), blood urea nitrogen (BUN), and creatinine (CRE) remained within physiological limits and showed no intergroup differences, excluding hepatic or renal toxicity (Figure S11A–D, Supporting Information). These findings confirm the excellent systemic biocompatibility of the GelSSO/PDA@SDF biomimetic niche in vivo.

## 3 Conclusion

In this study, to address the diminished abundance and activity of SCNs in the diabetic cranium, we developed an injectable GelSSO/PDA@SDF fluidic biomimetic niche, incorporating PDA@SDF NPs as an early‐phase and SSO‐grafted GelMA as a late‐phase signal amplification module, thereby enabling chronotaxic signal amplification to promote autonomous cranial regeneration. Upon in situ photocrosslinking, preferential and sustained release of PDA@SDF NPs initiated early‐phase signal amplification, characterized by recruitment of EPCs and MSCs via the SDF‐1α/CXCR4 axis, activation of the vascular niche that promotes angiogenesis via AKT signaling, and repolarization of macrophages toward a regenerative M2 phenotype. Subsequently, gradual degradation of the niche triggered steady release of SSO, further amplifying late‐phase signals to form a transcriptionally active osteogenic niche, which sustained MAPK/ERK‐mediated osteogenic differentiation of MSCs. In vivo experiments demonstrated that the fluidic biomimetic niche could be photopolymerized into a defect‐conforming structural niche that initially corrected local immune imbalance and endogenous progenitor recruitment, followed by enhanced neovascularization and lamellar bone formation, ultimately achieving vascularized regeneration in diabetic cranial defects. Collectively, by coupling chronotaxic signal amplification with structural adaptability, this injectable fluidic biomimetic niche presents a versatile and broadly translatable approach for in situ autonomous regeneration of diabetic bone defects and other poorly regenerative tissues.

## Conflict of Interest

The authors declare no conflict of interest.

## Author Contributions

Y.M. conceptualized, curated data, acquired funding, developed methodology, and wrote the original draft. Y.C. conceptualized, curated data, developed methodology, and wrote the original draft. R.F. performed formal analysis and curated data. P.Z. developed methodology and conducted formal analysis. H.Z. conceptualized, acquired funding, administered the project, supervised, and reviewed and edited the writing. P.Z. conceptualized, acquired funding, administered the project, supervised, and reviewed and edited the writing.

## Supporting information



Supporting Information

## Data Availability

The data that support the findings of this study are available from the corresponding author upon reasonable request.
