# Supplementary material for: Reinstating Niche Failure in Diabetic Cranial Defects via Chronotaxic Signal‐Amplifying Fluidic Biomimetic Hydrogel
Source: Adv Sci (Weinh). 2025 Oct 15;13(1):e16398. doi: 10.1002/advs.202516398 (PMC12766998; doi:10.1002/advs.202516398)
Supplement: Supplementary file 1 — Supporting Information [file ADVS-13-e16398-s001.docx]

**Supporting Information**

**Reinstating Niche Failure in Diabetic Cranial Defects via Chronotaxic Signal-Amplifying Fluidic Biomimetic Hydrogel**

*Yingji Mao, Yu Chen, Runlin Fan, Pengzhen Zhuang, Hongbo Zhang,* and Pinghui Zhou**

Y. Mao, R. Fan, P, Zhou

Department of Orthopedics, Anhui Province Key Laboratory of Tissue Transplantation, The First Affiliated Hospital of Bengbu Medical University, Bengbu 233004, China

E-mail: [phzhou@bbmu.edu.cn](mailto:phzhou@bbmu.edu.cn)

Y. Mao

Anhui Provincial Key Laboratory of Tumor Evolution and Intelligent Diagnosis and Treatment, Anhui Nerve Regeneration Technology and Medical New Materials Engineering Research Center, School of Life Sciences, Bengbu Medical University, Bengbu 233030, China

Y. Chen, P. Zhuang, H. Zhang

Pharmaceutical Sciences Laboratory, Faculty of Science and Engineering, Åbo Akademi University, Turku 20520, Finland

E-mail: [hongbo.zhang@abo.fi](mailto:hongbo.zhang@abo.fi)

H. Zhang

Turku Bioscience Centre, University of Turku and Åbo Akademi University, Turku, 20520, Finland

**Experimental Section**

**Synthesis and Characterization of PDA@SDF NPs**

PDA NPs were synthesized via oxidative self-polymerization of dopamine under alkaline conditions.^[1]^ Briefly, 200 mg of dopamine hydrochloride (Sigma-Aldrich, USA) was dissolved in 10 mL of Tris-HCl buffer (10 mM, pH 8.5, Sigma-Aldrich, USA) and gently stirred at 25 °C in the dark for 24 h, during which the solution gradually darkened from colorless. The resulting suspension was centrifuged at 14,000 rpm for 10 min and washed with ultrapure water to collect PDA NPs. For SDF-1α loading, 1 mg of lyophilized PDA NPs was ultrasonically dispersed in 1 mL of alkaline aqueous solution (pH 8.5) containing 10 μg of SDF-1α (Peprotech, USA), then gently shaken overnight at 4 °C. The suspension was ultracentrifuged to remove unbound SDF-1α, washed with precooled ultrapure water, and lyophilized to yield PDA@SDF NPs.

The surface morphology of PDA and PDA@SDF NPs was observed using SEM (Hitachi, Japan). Their nanostructure and elemental distribution were further characterized by TEM (Hitachi, Japan) equipped with EDS. The particle size was quantified from TEM images by measuring at least 100 randomly selected NPs using ImageJ software. The hydrodynamic size, its variation over 5 consecutive days in aqueous solution, and zeta potentials at pH 7.4 and 6.0 were determined at 25 °C via DLS using a Zetasizer Nano-ZS (Malvern, UK). The spectroscopic features of NPs were analyzed via FTIR (Thermo Fisher Scientific, USA) over 4000–400 cm^–1^, and UV-Vis spectroscopy (Shimadzu, Japan) between 200–800 nm.

**Synthesis of SSO**

The single-stranded 27-mer oligodeoxynucleotide, 5′-amino-(ACC CCC TCT)_3_-3′, was custom-synthesized (Sangon Biotech Co., China) as a tandem triplet of the human mitochondrial motif, with slight modification based on previous reports.^[2]^ To enhance nuclease resistance and permit covalent grafting, the oligodeoxynucleotide featured a fully phosphorothioated backbone and a 5′-terminal primary amine. Lyophilized SSO was reconstituted in nuclease-free water to 500 µM, aliquoted, and stored at –20 °C until use.

**Preparation of GelSSO Hydrogel Precursor**

The GelSSO hydrogel precursor was prepared through a series of sequential chemical modifications. First, GelMA was synthesized from porcine skin gelatin (Type A, Sigma-Aldrich, USA) by reaction with MA (Aladdin Industrial Co., China), following our earlier protocol.^[3]^ In brief, 20 g of gelatin pellets were dissolved in 200 mL of carbonate buffer (pH 9.0) at 50 °C to obtain a 10% (w/v) gelatin solution. Subsequently, 2 mL of MA was added dropwise at 0.2 mL min^–1^ and reacted for 3 h in the dark. The reaction was quenched with 100 mL of PBS (pH 7.4, Gibco, USA), and the residual MA was removed by centrifugation at 7500 rpm for 15 min. The supernatant was dialyzed (MWCO 8000, Spectra/Por®, USA) against ultrapure water at 37 °C for 2 days, lyophilized to produce porous white GelMA foam, and stored at –20 °C.

Subsequently, SSO was grafted onto GelMA via EDC/NHS (Aladdin Industrial Co., China) coupling chemistry. In short, 1 g of GelMA was dissolved in 16 mL of 50 mM 2-(N-morpholino) ethanesulfonic acid (MES, pH 5.5, Aladdin Industrial Co., China) buffer, followed by activation with 96 mg of EDC and 58 mg of NHS under continuous stirring for 30 min at room temperature. Then, 4 mL of MES buffer containing 500 µM SSO was added dropwise, and the mixture was reacted for 4 h. The resulting GelSSO was purified by centrifugation, dialysis, and lyophilization as described above. The grafting of MA and SSO onto GelMA was verified by ^1^H NMR (400 MHz, Bruker, Germany).

**Fabrication of GelSSO/PDA@SDF Biomimetic Niche**

To fabricate the fluidic biomimetic niche, 600 mg of GelSSO foam was completely dissolved in 6 mL of sterile PBS at 37 °C, supplemented with 0.5% (w/v) lithium phenyl-2,4,6-trimethylbenzoylphosphinate (Sigma-Aldrich, USA) as the photoinitiator. Then, 3 mg of PDA@SDF NPs were uniformly dispersed by vortexing and ultrasonication. The homogeneous precursor solution was sterilized through a 0.45 μm syringe filter and kept at 4 °C in the dark for immediate use. Analogous control precursors were prepared in parallel, including GelSSO alone, and GelMA either without or incorporating PDA@SDF NPs. For in-situ gelation, the pre-gel solution was injected into custom molds or cranial defects and photocrosslinked under 405 nm visible light to form a structural, biomimetic niche.

**Characterization of GelSSO/PDA@SDF Biomimetic Niche**

Gelation was qualitatively assessed using the tilted-vial method, which recorded gelation times. Macroscopic images of various hydrogels were captured using a digital camera (D3500, Nikon, Japan) after photocuring in circular molds. The microarchitecture and elemental composition were characterized by SEM coupled with EDS. Quantitative analysis of pore size and porosity was conducted using ImageJ software based on SEM micrographs. The static WCA was measured using a contact angle goniometer (DSA25S, DataPhysics Co., Germany) to assess the surface wettability. FTIR spectra were further used to compare the chemical structures among hydrogels.

The swelling and biodegradation were evaluated by immersing pre-weighed, lyophilized hydrogel discs (W_0_) in PBS containing collagenase type II (2 U mL^–1^) at 37 °C. At predetermined intervals within 48 h, samples were gently removed, surface moisture was blotted, and the swollen weight (W_t_) was recorded. The swelling ratio was calculated as (W_t_−W_0_)/W_0_, with the ESR defined as the value at which W_t_ plateaued. For biodegradation analysis, the same equation was applied after re-drying, with Wₜ representing the residual dry weight. The results were expressed as percentage residual mass over 28 days.

The rheological properties were investigated using a rotational rheometer (HAAKE MARS60, Thermo Fisher Scientific, USA) fitted with a 20 mm parallel-plate geometry and a 1 mm gap, operated at 25 °C. The shear viscosity of the precursor solution was measured while ramping the shear rate from 0.1 to 1000 s^–1^. After photocuring, oscillatory strain sweeps were executed at a constant frequency of 1 Hz across a strain range of 0.1% to 1000%, followed by a 20 min time sweep at 1% strain under the same frequency.

The compressive and adhesive performances were quantified using a universal tester (Instron 5969, 100 N load cell, Instron, USA) at room temperature. Cylindrical samples with 8 mm diameter and 5 mm thickness were compressed uniaxially at a constant strain rate of 1 mm/min until failure. The compressive modulus, compressive strength, failure strain, and toughness were extracted from the resulting curves. For lap-shear adhesion, the precursor solution was applied to the overlapping region of two freshly excised porcine skin slices, photocured, and pulled at 2 mm/min to detachment. The peak load divided by the overlap area was reported as adhesive strength. All mechanical parameters were summarized and compared using a radar chart.

The hydrogel precursor was transferred to a 1 mL syringe and extruded into a PBS-filled glass vial to assess injectability, with uninterrupted flow and filament continuity monitored photographically. An “S”-shaped trace was written with the precursor and photocrosslinked, followed by imaging at 0 and 1 h to evaluate geometric retention over time. The hydrogel precursor was photocured between a syringe plunger and a nitrile glove, and the bonded construct was suspended and filmed immediately and 1 h later to verify adhesive persistence.

In vitro release profiles were obtained by incubating hydrogel samples in PBS containing 0.1% (w/v) bovine serum albumin at 37 °C under gentle agitation. At predefined time points, the supernatant was collected and replenished with fresh buffer in equal volume. The concentration of SDF-1α was quantified using a commercial ELISA kit (R&D Systems, USA), while the released SSO was measured by an ultra-micro spectrophotometer (BioDrop, Biochrom, UK). The cumulative release percentage was calculated relative to the initial loading amount and plotted versus time.

**Cell Culture and Maintenance**

Murine macrophages (RAW 264.7, CL-0190, Procell, Wuhan, China), rBMSCs (CP-R131, Procell, Wuhan, China), and HUVECs (CRL-1730, ATCC, USA) were cultured under standard conditions. RAW264.7 cells and HUVECs were maintained in Dulbecco's modified Eagle medium-high glucose (4.5 g/L, Gibco, USA), whereas rBMSCs were grown in DMEM/nutrient mixture F-12 (Gibco, USA). All media were supplemented with 10% (v/v) fetal bovine serum (Gibco, USA) and 1% (v/v) penicillin-streptomycin (Gibco, USA). Cultures were incubated at 37 °C in a humidified 5% CO_2_ atmosphere, with medium refreshed every other day. At 80–90% confluence, cells were passaged at a 1:3 ratio using trypsin. Cells within passages 1–3 were used for subsequent assays. All cell lines were mycoplasma-free.

**In Vitro Cell-Hydrogel Interaction Modeling**

Two modes of cell-material interaction were employed based on experimental objectives. In the direct interaction mode (DIM), cells were seeded directly onto preformed hydrogels in a 24-well plate. In the indirect interaction mode (IIM), cells were inoculated in the lower chamber of a 24-well plate, while hydrogels were placed in a 0.4 μm-pore Transwell insert (Corning, USA) positioned above.

**Cytocompatibility Test**

HUVECs and rBMSCs were co-cultured with each hydrogel group at 4 × 10^4^ cells/well for 1, 4, and 7 days under the respective interaction models. Material biocompatibility was then comprehensively interrogated as detailed below.

To visualize cell viability, IIM-treated cells were stained using a live/dead reagent (Invitrogen, USA) composed of calcein-AM and ethidium homodimer-1 for 20 min at 37 °C. Fluorescent images of viable (green) and dead (red) cells were acquired under an inverted fluorescence microscope (Olympus, Japan), and merged images were analyzed using ImageJ software to calculate the viable cell ratio.

Before cytoskeletal staining, HUVECs and DIM-treated rBMSCs were fixed in paraformaldehyde, permeabilized with 0.1% Triton X-100 (Sigma-Aldrich, USA) for 10 min, and sequentially stained with 0.5% (v/v) rhodamine-phalloidin (Yeasen, China) for 30 min and 1% (v/v) 4′,6-diamidino-2-phenylindole (DAPI, Solarbio, China) for 5 min, protected from light. Red cytoskeletal morphology and blue nuclei were photographed by confocal laser scanning microscopy (ZEISS, Germany).

For cell proliferation, IIM cultures received fresh medium blended with 10% (v/v) CCK-8 reagent (Dojindo, Japan) and incubated for 4 h. Next, 100 µL of the reaction supernatant was transferred to a 96-well plate, and the absorbance at 450 nm was measured using a microplate reader (ThermoFisher Scientific, USA). LDH release into culture media was measured 24 h after IIM treatment using a LDH assay kit (Beyotime, China). Cells lysed with 0.1% Triton X-100 served as positive controls.

**Evaluation of Inflammatory Reversal Capacity**

RAW264.7 macrophages seeded at 8 × 10^4^ cells/well were primed with 100 ng/mL LPS (Sigma-Aldrich, USA) plus 20 ng/mL murine IFN-γ (Beyotime, China) for 24 h to induce M1 pro-inflammatory phenotype. Hydrogel-mediated immunomodulation was evaluated after 24 h of IIM treatment as described below.

For the ELISA assay, conditioned media were harvested, and concentrations of pro-inflammatory cytokines (TNF-α, IL-1β, IL-6) and anti-inflammatory cytokines (IL-4, IL-10) were quantified using commercial ELISA kits (Biosource Invitrogen, USA) according to the manufacturer's instructions.

For immunofluorescence staining, cells were fixed and treated with QuickBlock blocking buffer (Beyotime, China) comprising 0.1% Triton X-100 for 10 min. Subsequently, the cells were co-immunolabeled overnight at 4 °C with rat anti-CD68 (1:100, ab53444, Abcam, USA) coupled with rabbit anti-iNOS or anti-CD206 (1:100, ab178945 or ab300621, Abcam, USA), followed by Alexa Fluor-conjugated secondary antibodies: goat anti-rat 488 or 594 (1:1000, ab150157 or ab150160, Abcam, USA), and the corresponding goat anti-rabbit 594 or 488 (1:1000, ab150080 or ab150077, Abcam, USA) for 2 h at room temperature. Nuclei were counterstained with DAPI. Images were acquired on inverted fluorescence microscopy, and mean fluorescence intensity was semi-quantified using ImageJ software.

For ROS detection, cells were incubated with 10 µM DCFH-DA (Beyotime, China) at 37 °C for 30 min in the dark, washed with serum-free medium, counterstained with DAPI, and imaged under an inverted fluorescence microscope. Relative mean fluorescence intensity was analyzed using ImageJ software.

To further explore gene-level polarization markers, total RNA was extracted using TRIzol reagent (Invitrogen, USA) and quantified spectrophotometrically. Next, complementary DNA was synthesized using PrimeScript RT reagent kit (Takara, Japan), and qRT-PCR was performed on a QuantStudio 6 Flex system (Applied Biosystems, USA) with TB Green Premix Ex Taq II (Takara, Japan) and gene-specific primers (Table S1, Sangon Biotech Co., China). Relative gene level was calculated via the comparative CT (2−ΔΔCT) method, with glyceraldehyde-3-phosphate dehydrogenase (GAPDH) as the internal reference.

**Cell-Recruiting Potential Assessment**

The chemotactic capacity of hydrogels towards HUVECs and rBMSCs was studied via wound healing and Transwell migration assays. Around 90% confluent monolayers in 24-well plates were scratched and cultured under IIM in serum-free medium. At 0 and 24 h post-scratch, HUVECs labeled with calcein-AM and rBMSCs stained with 0.1% crystal violet (Solarbio, China) were imaged using an inverted fluorescence microscope. The scratch closure percentage was calculated using ImageJ software by comparing initial and residual scratch areas. In a 24-well Transwell system (8.0 μm pore size), 200 µL of serum-free medium packed with 2 × 10^4^ cells was settled into the upper chamber, while hydrogels were placed in the lower well submerged in 700 μL of serum-free medium. After 24 h, the polycarbonate membrane was fixed in paraformaldehyde and stained with 0.1% crystal violet for 10 min each. Non-migrated cells internal to the membrane were removed with cotton swabs, whereas migrated cells on the underside were imaged integrally under an optical microscope (Olympus, Japan), and five subfields were randomly selected for cell counting. To probe the recruitment mechanism, migrated cells were immunostained for CXCR4. The staining procedure was as described above, with rabbit anti-CXCR4 (AF5279, Affinity Biosciences, USA) and Alexa Fluor 594-conjugated goat anti-rabbit IgG used instead.

**In Vitro Vascularization Ability**

The pro-angiogenic potential of hydrogel-modulated HUVECs was evaluated using tube formation assays. First, 150 µL of cold, growth factor-reduced Matrigel (Corning, USA) was dispensed into 24-well plates and solidified at 37 °C. Hydrogels were pre-soaked in basal medium for 24 h to generate conditioned medium. Then, 3 × 10^4^ HUVECs seeded onto Matrigel layers were cultured with conditioned medium. After labeling with CellTrace Red CMTPX (Yeasen, China), network formation was imaged at 1 and 6 h under an inverted fluorescence microscope, and junction and mesh counts were quantified using ImageJ software.

Angiogenesis-related protein expression was analyzed after culturing 4 × 10^4^ HUVECs under DIM for 3 days. The primary and secondary antibodies utilized in immunofluorescence staining were rabbit anti-CD31 (AF6191, Affinity Biosciences, USA) and Alexa Fluor 594-conjugated goat anti-rabbit IgG, respectively. For Western blotting, total protein was extracted with radio immunoprecipitation assay buffer (Beyotime, China) and quantified by bicinchoninic acid protein assay kit (Beyotime, China). Equal protein loads were resolved by sodium dodecyl sulfate-polyacrylamide gel electrophoresis and blotted onto polyvinylidene fluoride membranes (Millipore, USA). After blocking with 5% skimmed milk in Tris-buffered saline-Tween for 1 h, membranes were probed overnight at 4 °C with rabbit antibodies against VEGFA, AKT, p-AKT, and GAPDH (1:1000, AF5131, AF6261, AF3263, AF7021, Affinity Biosciences, USA). Membranes were washed and incubated with horseradish peroxidase-conjugated goat anti-rabbit IgG (1:3000, S0001, Affinity Biosciences, USA) at room temperature for 1 h. Immunoreactive bands were visualized using an enhanced chemiluminescence substrate (Bio-Rad, USA) and semi-quantified densitometrically with Image Lab software (Bio-Rad, USA).

**In Vitro Osteogenesis Study**

To investigate the osteogenic effects of hydrogel intervention on rBMSCs, 4 × 10^4^ cells were exposed to either DIM or IIM for 24 h, followed by replacement with osteoinductive medium (Cyagen, China). On day 7, DIM-treated cells were stained with an ALP staining kit (Yeasen, China) and enzymatically quantified using ALP assay kit (Jiancheng Bioengineering Institute, China). Stained cultures were imaged under an optical microscope, and absorbance was measured at 405 nm to determine ALP activity. On day 21, extracellular calcium deposition in DIM-treated cells was observed by ARS staining (pH 4.2, Cyagen, China). After fixation and staining in the dark, red calcium nodules were photographed microscopically, then solubilized in 10% cetylpyridinium chloride for absorbance measurement at 420 nm. Immunofluorescence staining for OCN was performed on day 21 in DIM-treated cells using rabbit anti-OCN (DF12303, Affinity Biosciences, USA) and Alexa Fluor 488-conjugated goat anti-rabbit IgG.

On day 7, gene and protein levels related to osteogenesis were verified in IIM cultures by qRT-PCR and Western blotting, following the previously described protocols. Primer sequences for qRT-PCR are listed in Table S2. Western blotting employed rabbit antibodies against ALP, OCN, and RUNX2 (DF6225, DF12303, AF5186, Affinity Biosciences, USA), as well as MAPK pathway markers ERK and p-ERK (AF0155, AF1015, Affinity Biosciences, USA), with GAPDH as the loading control.

**In Vivo Diabetic Cranial Defect Modeling**

All animal procedures were approved by the Institutional Animal Care and Use Committee of Bengbu Medical University (Approval No. 2021272). To explore the efficacy of hydrogels in promoting diabetic bone regeneration, sixty male Sprague-Dawley rats (325 ± 25 g, Hefei Shushan Laboratory Animal Centre, China) were used to establish a STZ-induced type 1 diabetes mellitus model before cranial defect surgery.^[4]^ Briefly, after overnight fasting, rats received a single intraperitoneal injection of freshly prepared STZ (55 mg/kg, Sigma-Aldrich, USA) dissolved in 0.1 M sodium citrate buffer (pH 4.5, Solarbio, China). Fasting tail-vein glucose was monitored at 72 h post-injection and weekly thereafter, and rats displaying two consecutive values exceeding 16.7 mM were deemed diabetic.

Critical-size cranial defects were created under intraperitoneal anesthesia with 1% pentobarbital sodium (40 ± 10 mg/kg, Sigma-Aldrich, USA). After shaving and applying antisepsis, a midline scalp incision was made, and the soft tissues were retracted to expose the cranium. Bilateral full-thickness defects (5 mm diameter) were drilled into the parietal bones with a saline-cooled trephine. Defects were then filled with hydrogel precursor solutions and photocrosslinked in-situ, or left empty as controls (12 rats per group). Surgical incisions were sutured in layers, and prophylactic antibiotics were administered intramuscularly for 72 h postoperatively. At 1, 4, and 8 weeks after surgery, four rats per group were euthanized by pentobarbital overdose. Harvested cranial bones and major organs (heart, liver, spleen, lungs, kidneys) were fixed in 10% neutral buffered formalin, while serum samples isolated from whole blood were stored at –80 °C for further analysis.

**In Vivo Validation of Inflammatory Modulation and Cell Recruitment**

At 1 week postoperatively, hydrogel-driven inflammatory transition and cell accumulation within the defect were examined via immunofluorescence staining. Cranial samples were decalcified in 10% ethylenediaminetetraacetic acid (Solarbio, China) for 1 month with periodic solution replacement, followed by graded ethanol dehydration, xylene clearing, and paraffin embedding. Serial sections (5 μm) were collected for staining.

Macrophage polarization was assessed using co-immunostaining with anti-iNOS and anti-CD206. EPCs and MSCs were identified by immunostaining with anti-CD34 (AF5149, Affinity Biosciences, USA) and anti-CD90 (DF4804, Affinity Biosciences, USA), respectively. After tagging with species-specific fluorescent secondary antibodies and DAPI, sections were imaged on an inverted fluorescence microscope and analyzed semi-quantitatively using ImageJ software.

**In Vivo Bone Regeneration Evaluation**

Cranial samples harvested at 4 and 8 weeks postoperatively were scanned with a micro-CT system (SkyScan 1176, SkyScan, Belgium). Scanning was performed at 65 kV, 385 µA, with 18 μm resolution and a 1 mm aluminum filter. The data were imported into MIMICS 19.0 software (Materialise, Belgium) for threshold segmentation and 3D rendering. Bone regeneration within a cylindrical region matching the original defect dimensions was quantified as BV/TV and BMD.

Following micro-CT, samples were paraffin-embedded, sectioned, and subjected to H&E and Masson’s trichrome staining. Immunohistochemical detection of OCN was performed using 3,3′-diaminobenzidine (Solarbio, China), while expression of RUNX2 and CD31 were assessed by immunofluorescence staining.

**In Vivo Biocompatibility Evaluation**

At 8 weeks post-implantation, systemic biocompatibility was assessed histologically by H&E staining of harvested major organ sections. Concurrently, serum clinical chemistry assays were conducted to evaluate hepatic and renal functions, specifically measuring ALT, AST, BUN, and CRE.

**Statistical Analysis**

All experiments, unless otherwise indicated, were performed independently in triplicate. Numerical data were analyzed using GraphPad Prism 8.0 software (GraphPad Software Inc., USA) and are presented as mean ± standard deviation. Statistical differences among groups were determined by one-way analysis of variance followed by Tukey’s multiple comparison tests, with statistical significance defined as *p* < 0.05.

**References**

1. W. Cheng, X. Zeng, H. Chen, Z. Li, W. Zeng, L. Mei, Y. Zhao, *ACS Nano* **2019**, *13*, 8537.
2. a) Y. Mao, Y. Chen, W. Li, Y. Wang, J. Qiu, Y. Fu, P. Zhou, *Adv. Healthcare Mater.* **2022**, *11*, 2201457; b) G. Yang, M. Wan, Y. Zhang, L. Sun, R. Sun, D. Hu, Y. Yu, *Immunology* **2010**, *131*, 501.
3. Y. Wang, Y. Chen, T. Zhou, J. Li, N. Zhang, N. Liu, Y. Mao, *J. Nanobiotechnol.* **2024**, *22*, 702.
4. Y. Chen, Q. Liu, J. Guan, C. Zheng, S. Shi, W. Zheng, Y. Mao, *Mater. Today Bio* **2025**, *22*, 102079.

**Supplementary Figures**

**
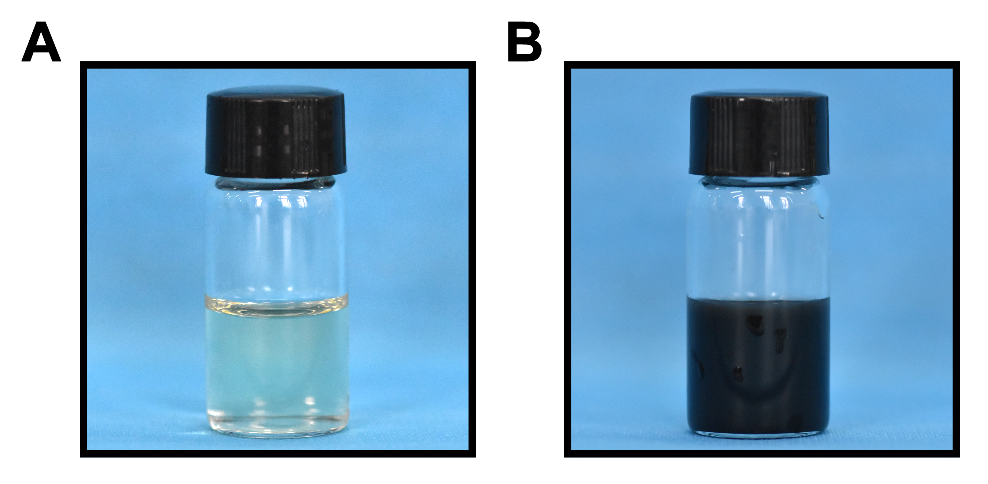
**

**Figure S1.** Dopamine autoxidation visualized during PDA synthesis. A) Dopamine hydrochloride solution. B) Polydopamine nanoparticle dispersion.

**
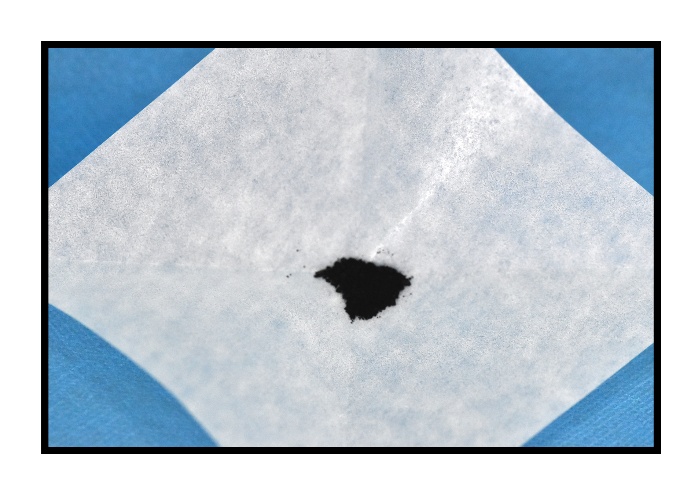
**

**Figure S2.** Lyophilized PDA@SDF nanoparticles after synthesis.

**
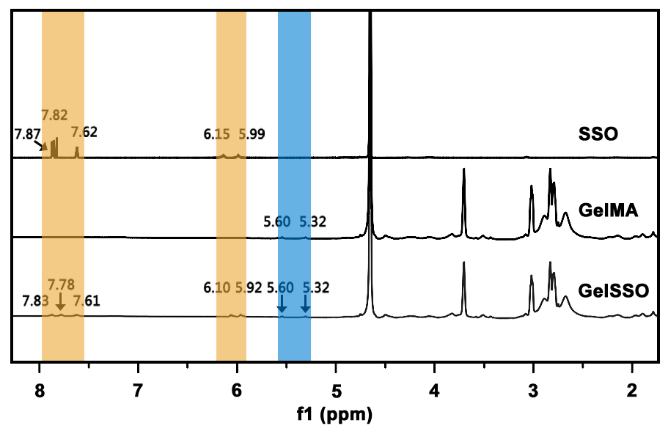
**

**Figure S3.** ^1^H nuclear magnetic resonance spectra of SSO, GelMA, and GelSSO.

**
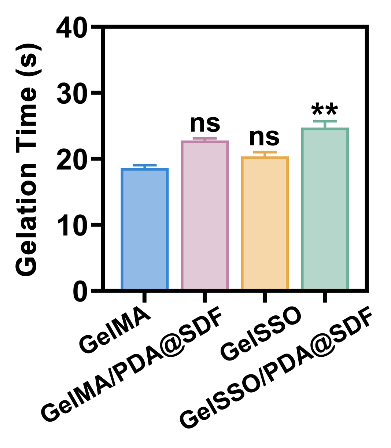
**

**Figure S4.** Gelation times for different hydrogels. (Data are presented as mean ± SD, n = 3, ns, not significant, ***p* < 0.01 compared with the GelMA group).

**
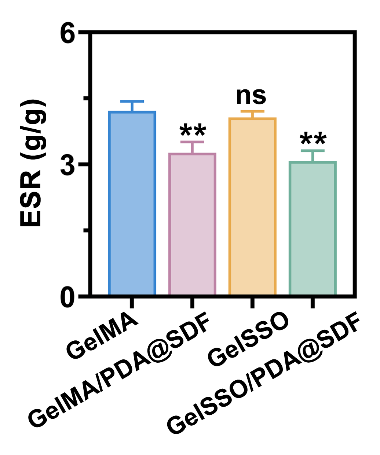
**

**Figure S5.** Equilibrium swelling ratio (ESR) of different hydrogels. (Data are presented as mean ± SD, n = 3, ns, not significant, ***p* < 0.01 compared with the GelMA group).


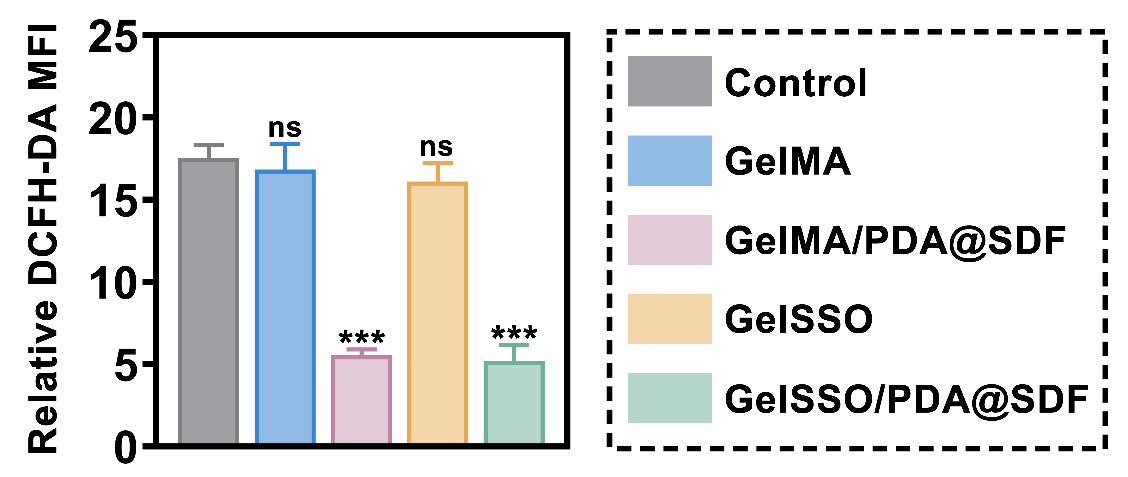


**Figure S6.** Semi-quantification of relative mean fluorescence intensity (MFI) for DCFH-DA. (Data are presented as mean ± SD, n = 3, ns, not significant, ****p* < 0.001 compared with the control group).

**
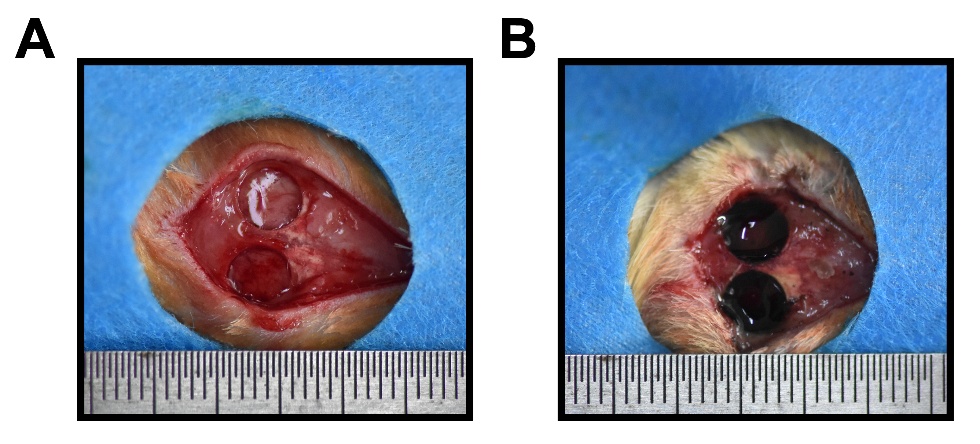
**

**Figure S7.** Representative intraoperative images from diabetic rat cranial defect modeling. A) Creation of bilateral 5 mm full-thickness defects. B) In-situ photocrosslinking of the GelSSO/PDA@SDF biomimetic niche immediately after injection.


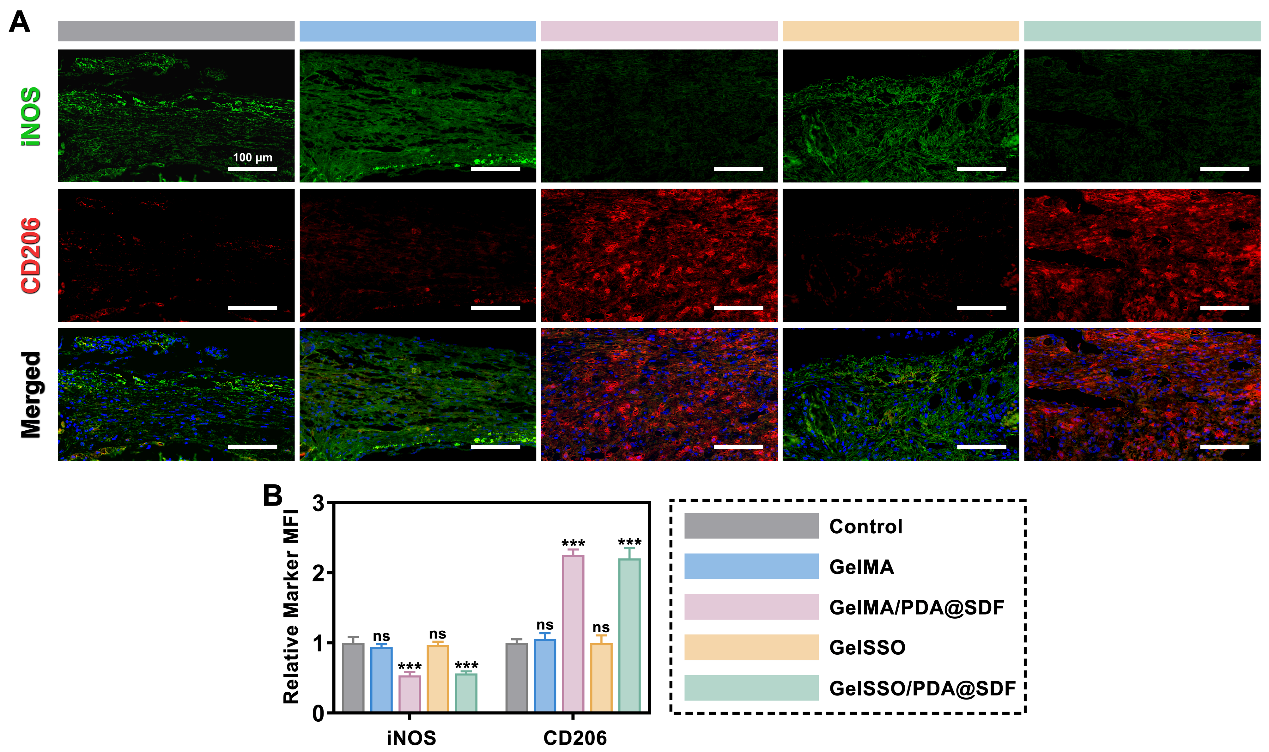


**Figure S8.** In vivo immunofluorescence analysis of macrophage polarization in diabetic cranial defects 1 week after biomimetic niche implantation. A) Representative images showing iNOS (green) and CD206 (red) expression in different hydrogel groups. B) Semi-quantification of relative mean fluorescence intensity (MFI) for iNOS and CD206. (Data are presented as mean ± SD, n = 4, ns, not significant, ****p* < 0.001 compared with the control group).


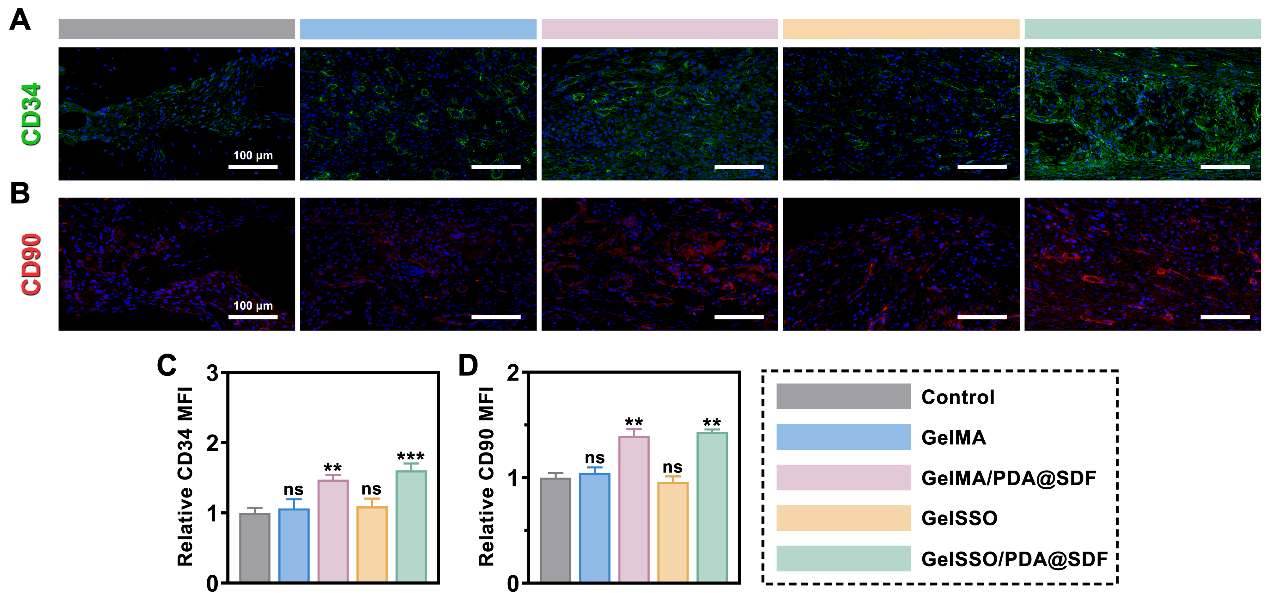


**Figure S9.** In vivo immunofluorescence analysis of endogenous cell recruitment in diabetic cranial defects 1 week after biomimetic niche implantation. A) Representative staining and C) semi-quantitative analysis of CD34-positive endothelial progenitor cells. B) Representative staining and D) semi-quantitative analysis of CD90-positive mesenchymal stem cells. (Data are presented as mean ± SD, n = 4, ns, not significant, ***p* < 0.01, ****p* < 0.001 compared with the control group).

**
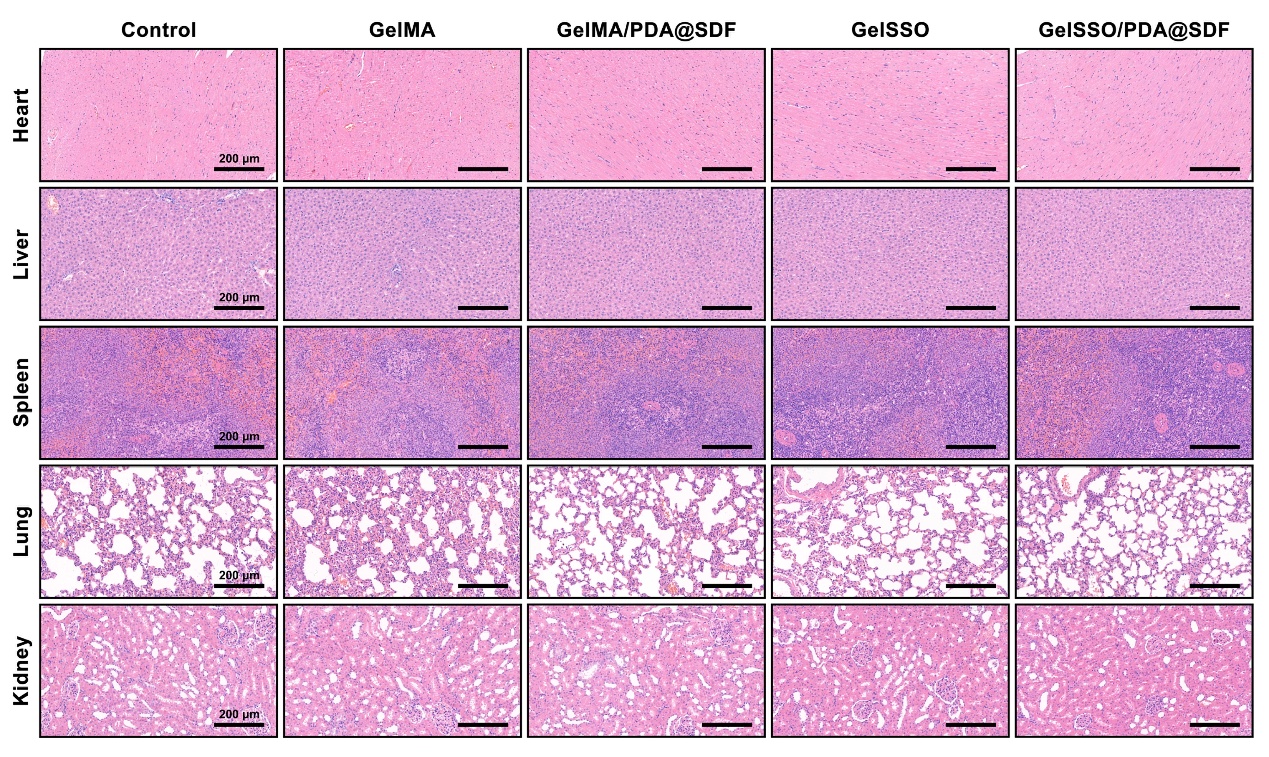
**

**Figure S10.** In vivo biocompatibility assessment of the biomimetic niche. Representative H&E staining of major organs (heart, liver, spleen, lung, and kidney) harvested 8 weeks postoperatively.

**
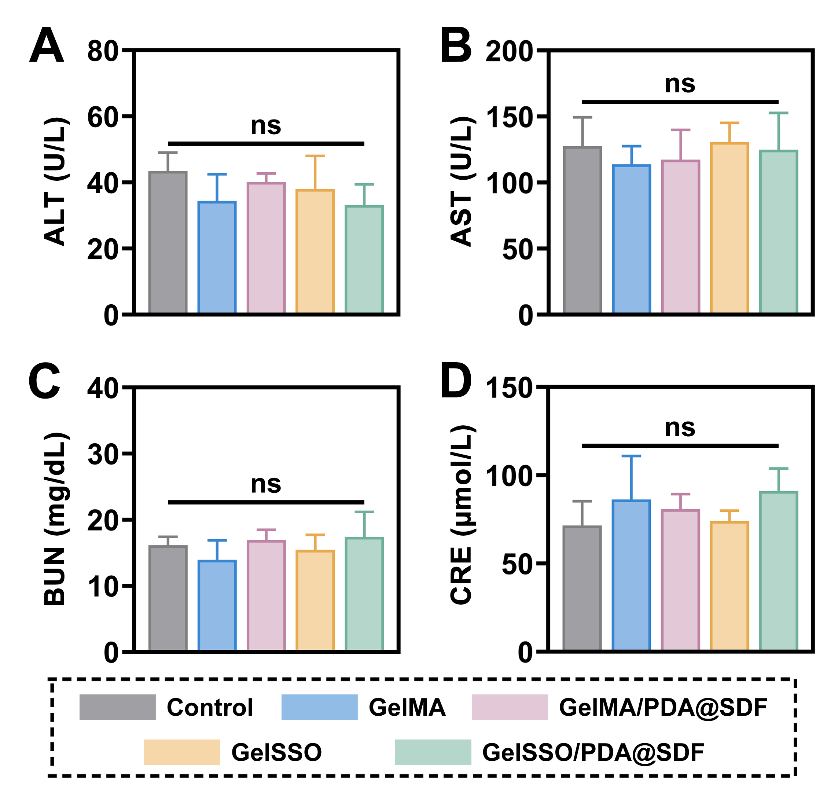
**

**Figure S11.** Systemic toxicity assessment of the biomimetic niche. Serum biochemical analysis of A) alanine aminotransferase (ALT), B) aspartate aminotransferase (AST), C) blood urea nitrogen (BUN), and D) creatinine (CRE) at 8 weeks postoperatively. (Data are presented as mean ± SD, n = 4, ns, not significant).

**Table S1.** qRT-PCR primer sequences for macrophage polarization markers in RAW264.7 cells.

| **Gene** | **Primer Sequences (5′–3′)** |
| --- | --- |
| iNOS | Forward: CATTGGAAGTGAAGCGTTTCG  Reverse: CAGCTGGGCTGTACAAACCTT |
| IL-1β | Forward: TCGAGGCCTAATAGGCTCATCT  Reverse: GCTGCTTCAGACACTTGCACAA |
| ARG1 | Forward: GAACACGGCAGTGGCTTTAAC  Reverse: TGCTTAGCTCTGTCTGCTTTGC |
| IL-10 | Forward: ACCTGCTCCACTGCCTTGCT  Reverse: GGTTGCCAAGCCTTATCGGA |
| GAPDH | Forward: TGTGGATGGCCCCTCTGGAA  Reverse: TCAGATGCCTGCTTCACCAC |

**Table S2.** qRT-PCR primer sequences for osteogenic markers in rBMSCs.

| **Gene** | **Primer Sequences (5′–3′)** |
| --- | --- |
| ALP | Forward: CGTCTCCATGGTGGATTATGCT  Reverse: CCCAGGCACAGTGGTCAAG |
| OCN | Forward: CATGAAGGCTTTGTCAGACT  Reverse: CTCTCTCTGCTCACTCTGCT |
| RUNX2 | Forward: TCTTCCCAAAGCCAGAGCG  Reverse: TGCCATTCGAGGTGGTCG |
| GAPDH | Forward: GGCAAGTTCAACGGCACAGT  Reverse: GCCAGTAGACTCCACGACAT |
